# A risk marker of tribasic hemagglutinin cleavage site in influenza A (H9N2) virus

**DOI:** 10.1038/s42003-020-01589-7

**Published:** 2021-01-15

**Authors:** Jiahao Zhang, Kaixiong Ma, Bo Li, Yiqun Chen, Ziwen Qiu, Jinchao Xing, Jinyu Huang, Chen Hu, Yifan Huang, Huanan Li, Dingxiang Liu, Ming Liao, Wenbao Qi

**Affiliations:** 1 College of Veterinary Medicine, South China Agricultural University, 510642 Guangzhou, China; 2 National Avian Influenza Para-Reference Laboratory, 510642 Guangzhou, China; 3 National and Regional Joint Engineering Laboratory for Medicament of Zoonoses Prevention and Control, National Development and Reform Commission of the People’s Republic of China, 510642 Guangzhou, China; 4 Key Laboratory of Zoonoses, Ministry of Agricultural and Rural Affairs of the People’s Republic of China, 510642 Guangzhou, China; 5 Guangdong Province Key Laboratory of Microbial Signals and Disease Control and Integrative Microbiology Research Centre, 510642 Guangzhou, China; 6 Guangdong Laboratory for Lingnan Modern Agriculture, 510642 Guangzhou, China; 7 Key Laboratory of Animal Vaccine Development, Ministry of Agricultural and Rural Affairs of the People’s Republic of China, 510642 Guangzhou, China; 8 Key Laboratory of Zoonoses Prevention and Control of Guangdong Province, 510642 Guangzhou, China

**Keywords:** Influenza virus, Viral pathogenesis, Viral evolution

## Abstract

Low pathogenic avian influenza A(H9N2) virus is endemic worldwide and continually recruit internal genes to generate human-infecting H5N1, H5N6, H7N9, and H10N8 influenza variants. Here we show that hemagglutinin cleavage sites (HACS) of H9N2 viruses tended to mutate towards hydrophilic via evolutionary transition, and the tribasic HACS were found at high prevalence in Asia and the Middle East. Our finding suggested that the tribasic H9N2 viruses increased the viral replication, stability, pathogenicity and transmission in chickens and the virulence of mice compared to the monobasic H9N2 viruses. Notably, the enlarged stem-loop structures of HACS in the RNA region were found in the increasing tribasic H9N2 viruses. The enlarged HACS RNA secondary structures of H9N2 viruses did not influence the viral replication but accelerated the frequency of nucleotide insertion in HACS. With the prevailing tendency of the tribasic H9N2 viruses, the tribasic HACS in H9N2 viruses should be paid more attention.

## Abstract (web summary)

Jiahao Zhang, Kaixiong Ma, and Bo Li et al. identify a variant of the H9N2 avian influenza virus within the hemagglutinin cleavage site required for viral infection. They show that this variant leads to higher viral replication in cells and more virulent disease in both chickens and mice.

## Introduction

The influenza A (H9N2) viruses were enzootic in birds and undergone continuous evolution in North America, Europe, South Asia and the Middle East. The Eurasian lineage formed three distinct sublineages represented by their prototype strains: A/quail/HongKong/G1/97 (G1-like), A/duck/HongKong/Y280/97 (Y280-like), and A/chicken/HongKong/Y439/97 (Y439-like)^1–4^. Although frequently updated commercial vaccines were used in areas of endemicity, the influenza A (H9N2) viruses continued to persist in avian and wild birds. The broad prevalence of the influenza A (H9N2) virus in birds naturally increased the risk of transmission to mammals, including dogs, pigs, and humans^5–8^. From 2013 to 2018, 27 human cases were laboratory-confirmed in China, Egypt, and Bangladesh as influenza A(H9N2) viruses, while only 13 cases were reported prior to 2012^5,7,9–12^, suggesting an increasing threat to human health. Strikingly, influenza A(H9N2) virus had continuously contributed to some zoonotic spillover events via the reassortment of cross-species variants, including the H5N1 influenza virus in HongKong in 1997, the H7N9 and H10N8 influenza viruses in 2013, and the H5N6 influenza virus in 2015^13–17^, posing public health concerns.

An essential step in infection by influenza A viruses is the proteolytic processing of the trimeric hemagglutinin cleavage site (HACS)^18^. For infection to occur, the HA0 precursor must be cleaved into the HA1 and HA2 subunits, thereby revealing a fusion peptide for membrane fusion^19^. Although the infection of H9N2 viruses is usually mild and localized to the respiratory and intestinal tracts due to the restriction of the proteases, the cleavage sites of distinct H9N2 lineages present are with the continuing evolution^7,20–22^. H9N2 viruses varied remarkably in the amino acid at the HACS. The nomenclature of the monobasic, dibasic, and tribasic influenza viruses was designated previously, with basic amino acids occurring once, twice, and three times, respectively. Viruses with the tribasic PAKSKR/G-motif and dibasic PARSSR/G-motif, PAKSSR, and PSRSSR/G-motif viruses in the Y280-sublineages and G1-sublineages had been found at high prevalence in Asia and the Middle East in birds and humans. The monobasic H9N2 viruses bearing one basic amino acid residue at the P1 position were frequently found in the Y439-sublineage and North America-lineage in wild birds; however, there had been no human infections. Thus, the main question is which HACS motifs in distinct lineages confer a replicative advantage to the virus in chickens and mammals and pose an increasing threat to humans.

The H5 and H7 subtype influenza viruses can evolve from low pathogenic (LP) to highly pathogenic (HP) in natural reservoirs, in which virulence can be increased in birds and can then posed threats to humans^14,23,24^. A previous study suggested that the enlarged stem-loop structures of HACS accelerated the multiple adenine and/or guanine insertions required to create codons for basic amino acids^24^. Although the H9N2 viruses were LP and did not possess a polybasic cleavage sites, some carry a motif similar to the cleavage sites were found in the HP H5N1 and H7N3 viruses^25–27^, indicative of the potential to acquire a polybasic site. In this study, we analyzed the genetic evolution and the structures of HACS among distinct H9N2 lineages, and introduced a scatter plot tool to show the selection and avoidance of certain predominant HACS mutations. Then, we evaluated the viral replication, stability, cleavage efficiency, and virulence in chickens and mice of H9N2 viruses bearing different cleavage site motifs. In addition, the correlation between the frequency of the nucleotide insertion and the size and locations of the stem-loop structures of different HACS among H9N2 viruses was assessed.

## Results

### Bioinformatic analysis of the cleavage sites of global influenza A (H9N2) viruses

**Fig. 1:**
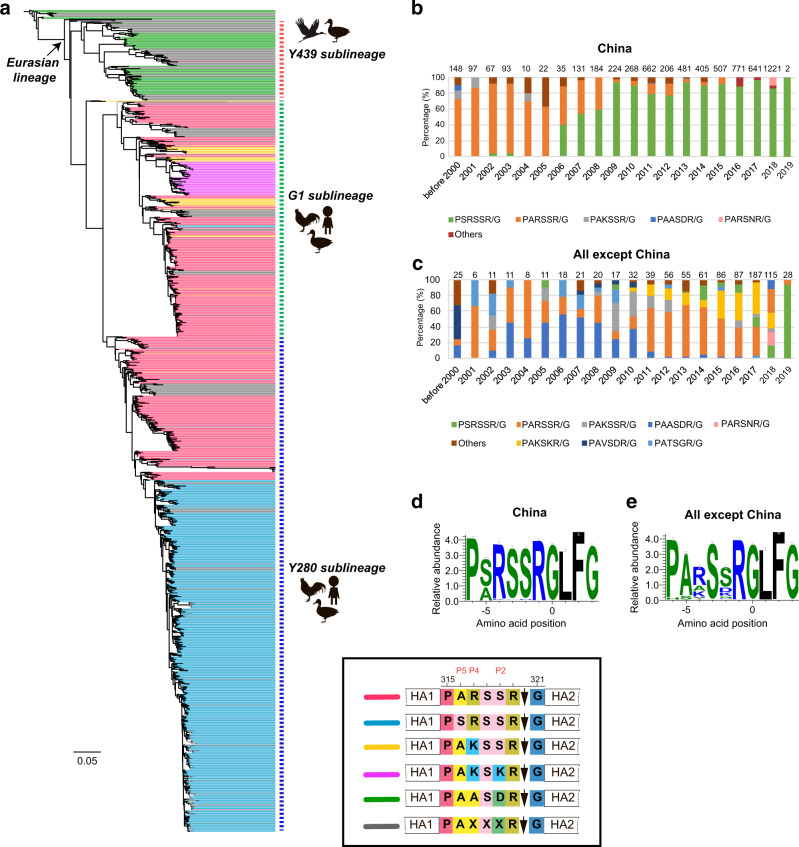
Evolutionary history of HACS of the influenza A(H9N2) viruses. **a** Maximum likelihood (ML) phylogeny of the H9N2 influenza virus HA gene. The HA gene sequences from all available H9N2 viruses were downloaded from GISAID’s EpiFlu Database for phylogenetic analysis. The ML tree was inferred with the RAxML software under the GTRGAMMA model with 1,000 bootstraps. References H9N2 viruses from each HACS motif are denoted by different colors. The distribution of different HACS motif of influenza A (H9N2) viruses from GISAID’s EpiFlu and GenBank Database. The branch length is scaled according to the numbers of substitutions per site (subs/site). **b** represents the distribution of the amino acids of HACS except for China, and **c** represents the amino acids of HACS in China. Different colored bars represent multiple HACS motifs of influenza A(H9N2) viruses. Amino acids frequency of all available HACS of influenza A(H9N2) viruses. Frequencies of each position, from P6 to P4’ of the HACS are illustrated using WebLogo 3.4 (http://weblogo.threeplusone.com/). **d** represents China, and **e** represents all countries except for China.

**Fig. 2:**
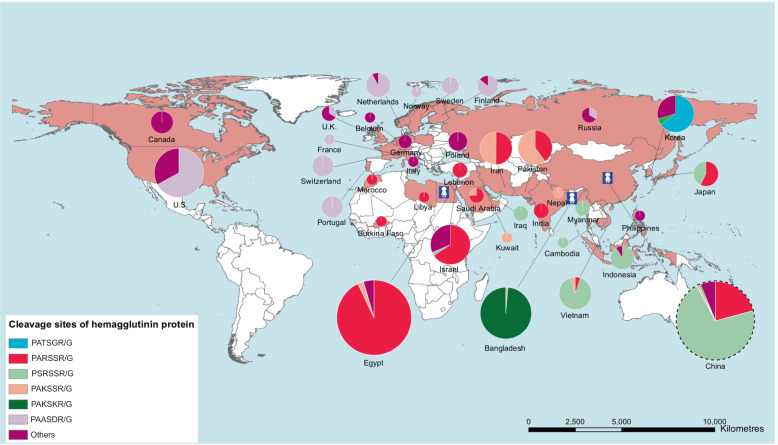
Geographic distributions of HACS of global influenza A(H9N2) viruses. Locations of human infections with H9N2 viruses are indicated by the blue cartoon figure. In the pie charts, the size represents the number of H9N2 isolates, and the colors represents different motifs of HA cleavage sites (HACS). The size of the dotted pie chart does not represent the number of H9N2 isolates in China owing to a large quantity of strains. Data are available from the GISAID’s EpiFlu Database, GenBank Database, the World Health Organization, the World Organisation for Animal Health, and the National Health and Family Planning Commission of the People’s Republic of China. The map was designed using ArcGIS Desktop 10.4 software (http://www.esri.com/software/arcgis/arcgis-for-desktop/).

To better understand the evolution, prevalence, and molecular features of the cleavage sites of the global influenza A (H9N2) viruses, hemagglutinin (HA) sequences were studied from a genetic perspective by performing multiple sequence alignment and phylogenetic analysis. With the flexibility of the P2, P4, and P5 positions in the HACS, the cleavage sites showed variability in different clades as depicted by WebLogo (Fig. 1a, d, e). The amino acid sequences of the cleavage sites of the HA1/HA2 junction possessed 5 main cleavage motifs, including PARSSR/G-motif, PSRSSR/G-motif, PAKSSR/G-motif, PAKSKR/G-motif, and PAASDR/G-motif (Fig. 2). We found that a steep increase in the number of PSRSSR/G-motifs occurred in China after 2006 (Fig. 1b,d and Fig. 2). Soon afterwards, the PSRSSR/G-motif had replaced the PARSSR/G-motif as a dominant cleavage motif of Y280-like H9N2 viruses in chickens and humans. The PARSSR/G and PAKSSR/G-motifs were widely prevalent in some countries, mainly including China, Iran, Pakistan, Bangladesh, Kuwait, and Saudi Arabia before 2010 (Fig. 2). Of note, a tribasic PAKSKR/G-motif of G1-like virus occurred in Bangladesh since 2010, with PAKSKR/G-motif viruses occurring in almost all H9N2 viruses during 2011-2019 (Fig. 1c,e). Notably, the PAASDR/G-motif of H9N2 viruses had been circulating in most European and North American countries. The HACS of Y439-like viruses was more polymerous in migratory birds, mainly including the PATSGR/G, PAASDR/G, and PAASYR/G motifs.

### The P2 and P4 positions at cleavage sites of H9N2 viruses influence the viral replication and cleavage efficiency

**Fig. 3:**
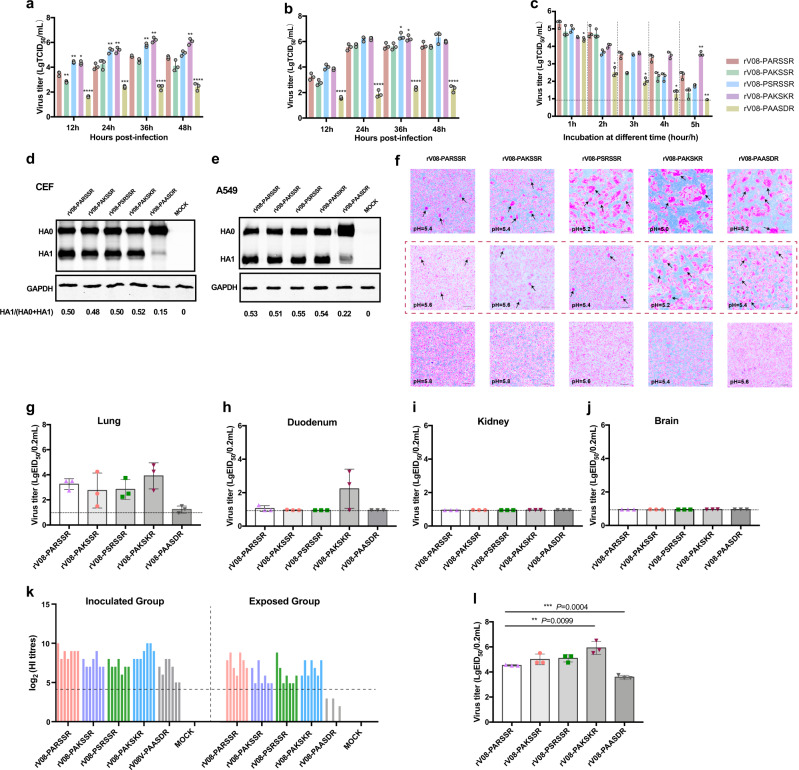
The biological characteristics of influenza A(H9N2) in vivo and in vitro. Growth curves after inoculation of each virus at a multiplicity of infection (MOI) of 0.001 into (**a**) MDCK cells and at an MOI of 0.001 into (**b**) CEF cells. Each point on the curve is the mean ± standard deviation from three independent experiments. The independent-samples *t* test was used for analysis. **c** Thermo stability of the influenza A(H9N2) viruses. The influenza A(H9N2) viruses were incubated for 1, 2, 3, 4, and 5 h at 50 °C. The titers of heat-treated recombination viruses were determined by TCID_50_ assay in MDCK cells. Western blots of CEF (**d**) and A549 (**e**) cell cultures inoculated with five influenza A(H9N2) virus at an MOI of 0.01 in the presence of 1 *μ*g/ml trypsin for 24 h. **f** Syncytia formation induced by five influenza A(H9N2) viruses in Vero cells. Cultures of Vero cells were inoculated with H9N2 viruses at MOI of 1 for 16 h, followed by incubation with 10 *μ*g/ml of TPCK-trypsin for 15 min to cleave expressed HA0. To induce syncytia formation, Vero cells were incubated for 5 min with fusion buffers of pH values ranging from 5.0 to 5.8, with 0.2 unit increments, and were visualized 3 h later. Representative images at pH values of 5.0 to 5.8 derived from three replicates are shown. Five groups of ten 5-week-old SPF chickens were infected intranasally with 10^6^ EID_50_/200 μL of each virus. **g** Viral titers in the lungs of three inoculated chickens. **h** Viral titers in the duodenum of three inoculated chickens. **i** Viral titers in the kidney of three inoculated chickens. **j** Viral titers in the brain of three inoculated chickens. **k** Antibody titers of inoculated and exposed chickens at 14 dpi. An HI titer ≥1:16 was considered seroconversion. Five groups of three female BALB/c mice were intranasally inoculated with 10^6^ EID_50_/50 μL of the H9N2 viruses. Three mice from each group were euthanized at 4 dpi. Viral titers in the mouse lungs (**l**) were shown. The dashed lines represent the detection limit. The independent-samples *t* test was used for analysis.

A reverse genetic system of an H9N2 virus, A/chicken/Guangdong/V/2008 (rV08-PARSSR), with an HA-PARSSR/G cleavage motif, was established previously^28^. Different H9N2 viruses with monobasic, dibasic, and tribasic cleavage sites were generated by site-directed mutagenesis (Supplementary Data 1), including the PSRSSR/G, PAKSSR/G, PAKSKR/G, and PAASDR/G cleavage motifs. To compare the replicative abilities of the H9N2 viruses, we inoculated Madin-Darby canine kidney (MDCK) and chicken embryo fibraoblasts (CEF) cells with a multiplicity of infection (MOI) of 0.001 and 0.001, respectively. Then, we determined the viral titers of supernatants at 12, 24, 36, and 48 h post-infection (hpi) after infection. In MDCK cells, the growth rate of rV08-PSRSSR and rV08-PAKSKR were higher than that of parental virus (Fig. 3a; Supplementary Data 2). By contrast, the replication of rV08-PAASDR markedly decreased compared with that of the parental virus at each time point (Fig. 3a; Supplementary Data 2). In CEF cells, the replicative ability of the rV08-PAKSSR, rV08-PSRSSR, and rV08-PAKSKR was not significant when compared with that of the parental virus. However, the rV08-PAASDR grew to a lower titer at each time point (Fig. 3b; Supplementary Data 3). To determine the influence of the H9N2 viruses on the activation of the cleavage of the HA precursor HA0, a Western blot analysis was performed with virus-infected A549 and CEF cell cultures inoculated with H9N2 viruses at an MOI of 0.01 in the presence of 1 μg/ml tosylsulfonyl phenylalanyl chloromethyl ketone (TPCK)-treated trypsin for 24 h. Our results showed that the cleavage efficiencies of CEF-grown and A549-grown rV08-PARSSR, rV08-PAKSSR, rV08-PSRSSR, and rV08-PAKSKR were similar, while the CEF-grown and A549-grown HA0 precursors of rV08-PAASDR could not be efficiently cleaved, although bearing supplemental TPCK-treated trypsin (Fig. 3d, e; Supplementary Figs. 1–2). In addition, we determined the cleavage efficiency of H9N2 viruses at different concentrations of TPCK-treated trypsin in the CEF cells (Supplementary Figs. 1–2), and we found that the rV08-PSRSSR and rV08-PAKSKR displayed no significant cleavage efficiency than did the parental virus. These results suggested that the P2 and P4 positions of HACS was crucial to viral replication and cleavage efficiency.

### The P2 and P4 positions at cleavage sites of H9N2 viruses influence the pH stability and thermostability

Here, we investigated the influence of the HA cleavage mutations on thermal stability. In contrast to the other H9N2 viruses, the rV08-PAKSKR virus displayed a significantly increased infectious titer after incubation at 5 hpi compared with the parental H9N2 viruses (Fig. 3c; Supplementary Data 4). In contrast, the rV08-PAASDR virus were observed to be less stable than the parental virus at each time point (Fig. 3c; Supplementary Data 4). No significant difference was observed for the rV08-PAKSSR and rV08-PSRSSR viruses when compared to the parental virus (Fig. 3c; Supplementary Data 4). These results demonstrated that the tribasic H9N2 virus was more stable; however, the monobasic H9N2 virus was less stable than the other cleavage motif viruses. To assess the acid stability of the five H9N2 viruses, a syncytium formation assay was performed to measure the pH threshold required for HA-mediated cell-to-cell fusion. Vero cells were inoculated with the five H9N2 viruses and exposed to trypsin to cleave and activate the HA; subsequently, the cell culture was acidified with a pH gradient of pH 5.0, 5.2, 5.4, 5.6, and 5.8. Visual inspection of the cell cultures for the presence of syncytia was used to determine the pH threshold triggering the conformational change and subsequent membrane fusion. The pH values at which cell-to-cell fusion was triggered by PARSSR/G, PAKSSR/G, PSRSSR/G, and PAASDR/G-motif viruses were 5.6, 5.6, 5.4, and 5.4, respectively (Fig. 3f). However, the pH value at which cell-to-cell fusion of the PAKSKR/G-motif virus occurred was 5.2 (Fig. 3f), indicating that the P2 and P4 position at cleavage site affect the cell-to-cell fusion activation of H9N2 viruses.

### The P2 and P4 positions at cleavage site influence the virulence and transmissibility of H9N2 virus in chickens

To determine which cleavage site mutations abolished the virulence and transmissibility in chickens, ten 5-week-old White Leghorn specific-pathogen-free (SPF) chickens were intranasally inoculated with 10^6^ 50% egg infection dose (EID_50_)/200 μl of H9N2 viruses. After 24 h, the ten uninfected chickens were housed with the inoculated groups. All of the H9N2 viruses could be detected in lung samples from the inoculated chickens except with the rV08-PAASDR group (Fig. 3g; Supplementary Data 5). Remarkably, the inoculated and exposed chickens of  rV08-PAKSKR group showed elevated virus output from the duodenum samples (Fig. 3h; Supplementary Data 5). The viral titers of rV08-PAKSKR could be detected in the duodenum samples of inoculated and exposed chickens, with an average of the ~10^2.2^ log_10_EID_50_/200 μl and ~10^1.5^ log_10_EID_50_/200 μl, respectively (Supplementary Data 5). No virus was detected in the kidney and brain samples of inoculated chickens of each group (Fig. 3i, j; Supplementary Data 5).

In addition, virus shedding was also detected in tracheal and cloacal swab speciments at 3, 5, 7, 9, and 11 day post-inoculation (dpi). The inoculated chickens in the rV08-PARSSR, rV08-PAKSSR, rV08-PSRSSR, and rV08-PAASDR group shed viruses at 3 and 5 dpi; however, virus shedding in the rV08-PAKSKR group lasted for 7 dpi (Supplementary Data 6). The virus in the trachea of all inoculated chickens exhibited robust shedding at 3 dpi and 5 dpi, whereas the cloacal virus shedding showed different time points of each group. The rV08-PAASDR virus shedding from the trachea only lasted for 3 days; however, the other groups shed comparable levels of infectious virus from the trachea until 7 dpi (Supplementary Data 6). Notably, the rV08-PAKSKR virus showed cloacal virus shedding in all of the inoculated chickens at 3 dpi and 5 dpi, but the other H9N2 viruses were shed via the cloacal route by less than half of the inoculated chickens at each time point (Supplementary Data 6). The transmission study showed that the exposed chickens in the rV08-PAASDR group failed to shed viruses and exhibited no seroconversion (Fig. 3k; Supplementary Data 7). Remarkably, the parental and other recombinant viruses were able to transmit to exposed chickens. Virus shedding of the exposed chickens in the rV08-PARSSR, rV08-PAKSSR, and rV08-PSRSSR groups mostly peaked from 5 to 7 dpi and decreased after 9 dpi. In the rV08-PSRSSR group, however, 4/7 exposed chickens had detectable trachea virus shedding at 9 dpi, while the parental virus was not shed (Supplementary Data 6). Of the rV08-PAKSKR virus, there appeared that the exposed chickens shed virus at the highest level and for the longest duration. The exposed chickens in this group shed virus via cloacal and tracheal routes at 5, 7 and 9 dpi, and the number of chickens shedding in the rV08-PAKSKR virus-infected group was more than that of the rV08-PAASDR H9N2 viruses at 7 and 9 dpi (Supplementary Data 6). Collectively, the results showed that the H9N2 virus possessing the monobasic PAASDR/G cleavage motif did not cause a productive infection and transmission in chickens; however, the tribasic H9N2 virus conferred a greater severity of infection and transmissibility, more prolonged shedding, and digestive viral spread in chickens.

### The tribasic H9N2 virus enhanced the pathogenicity in mice

To investigate the effects of H9N2 viruses containing different cleavage sites in mammals, 10^6^ EID_50_/50 μl of each virus was intranasally inoculated into 4-week-old female BALB/c mice. All five H9N2 viruses could replicate efficiently in the lungs of the inoculated mice by 4 dpi. There was no significance in the titers of the rV08-PAKSSR viruses compared with those of the parental rV08-PARSSR virus. However, the rV08-PAKSKR (0.001 < *p* < 0.01) in the lungs was significantly higher than the parental rV08-PARSSR virus at 4 dpi, while the virus titers of rV08-PAASDR virus in the lungs was lower than that of the rV08-PARSSR virus (0.0001 < *p* < 0.001) (Fig. 3l; Supplementary Data 5). No H9N2 viruses were detected in the brains infected with the rV08-PARSSR, rV08-PAKSSR, and rV08-PAASDR viruses. However, the H9N2 viruses had been detected in one  mice brain infected with the rV08-PSRSSR and rV08-PAKSKR virus (Supplementary Data 5).

### The enlarged stem-loop RNA secondary structure of the cleavage sites in H9N2 virus accelerated the nucleotide insertions in the HACS

**Fig. 4:**
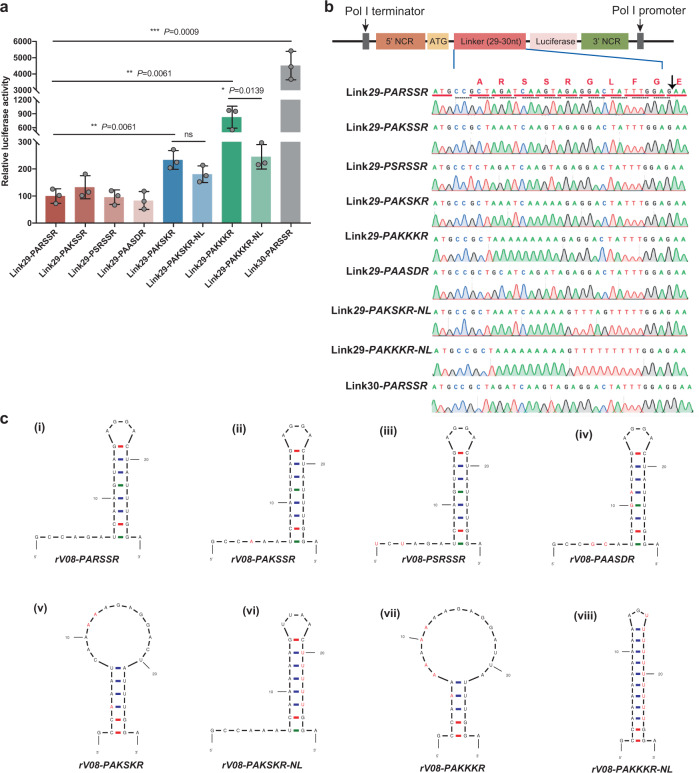
Quickfold-predicted RNA structures of the HA cleavage sites and luciferase activity in DF-1 cells. DF-1 cells were transfected with plasmid. The predicted RNA secondary structures of each HA cleavage site motif and amino acids corresponding to each codon are shown. Nucleotides different from the PARSSR/G-motif are shown in red. **a** Luciferase activities were expressed relative to the Link30-PARSSR plasmid and compared among the reporter plasmids containing the different linkers, including Link28-PARSSR, Link29-PARSSR, Link29-PSRSSR, Link29-PAKSSR, Link29-PAKSKR, Link29-PAASDR, Link29-PAKKKR, Link29-PAKSKR-NL, and Link29-PAKKKR-NL. Each point on the curve is the mean ± standard deviation from three independent experiments. The independent-samples *t* test was used for analysis. **b** The linker 29 and 30 polynucleotides were inserted between a start codon and the firefly luciferase gene lacking its start codon. **c** Quickfold-prediction RNA structures of the HACS sequences. The predicted RNA secondary structure of rV08-PARSSR (i), rV08-PAKSSR (ii), rV08-PSRSSR (iii), rV08-PAASDR (iv), rV08-PAKSKR (v), rV08-PAKSKR-NL (vi), rV08-PAKKKR (vii), and rV08-PAKKKR-NL (viii) are shown. Nucleotides different from the parental rV08-PARSSR are shown in red.

To evaluate the frequency of the nucleotide insertions in the distinct HACS regions of H9N2 viruses, we comprehensively compared the stem-loop structures of different cleavage site regions in H5, H7, and H9 subtype viruses. Compared with low pathogenic H5 and H7 subtype precursors, the well-positioned loop structure occurred less frequency in the HACS of most H9 subtype viruses, and the locations and sizes of the stem-loop structure of distinct cleavage site regions in H9N2 viruses were not significant enough (Supplementary Figs. 3–5). The HACS of most H9N2 viruses usually contain one small stem-loop structure consisting of six nucleotides, which include the successive adenines or guanines (5’-AGGGGA-3’). However, the locations of the stem-loop structure changed when the cleavage sites were varied, resulting in two or three small discontinuous stem-loop structures (Fig. 4c; Supplementary Figs. 6–8). Interestingly, the stem-loop structure of one tribasic cleavage site, the PAKSKR/G motif circulating in Bangladesh, tended to be larger than those of the HACS of H9N2 viruses (Fig. 4c; Supplementary Figs. 6–8). This large stem-loop structure of the HACS consisted of ten nucleotides and fully included the codons for the successive adenine or guanine (5’-AAAAAGAGGA-3’).

To assess the correlation between the frequency of the nucleotide insertions and the stem-loop structures of distinct HACS, a reporter assay^24^ was established to detect the nucleotide insertions in the RNA sequences encoding the amino acids in the HACS (containing 29 nucleotides) of the rV08-PARSSR virus (Supplementary Fig. 9). In our reporter plasmids, the firefly luciferase gene lacking its start codon was inserted downstream of the start codon, and the firefly luciferase was expressed when nucleotides were inserted into the cleavage site regions to make the sequence in frame with the open reading frame (ORF) (Fig. 4b). To test this system, the chicken fibroblast (DF-1) cells were transfected with the Link29-PARSSR and Link30-PARSSR plasmids, and the luciferase activities were measured. We observed that a high level of luciferase activity upon transfection with the Link30-PARSSR compared with Link29-PARSSR (Fig. 4a; Supplementary Data 8). Next, we constructed the reporter plasmids with different cleavage site motifs (i.e., PARSSR/G, PSRSSR/G, PAKSSR/G, PAKSKR/G, and PAASDR/G) and compared the luciferase activities. Our results showed that the Link29-PAKSKR significantly enhanced the luciferase expression compared with the Link29-PARSSR plasmid, while the luciferase activities did not increase when transfected with the other reporter plasmids (Fig. 4a; Supplementary Data 8). We then constructed the Link29-PAKKKR plasmid, which further enlarged the stem-loop structure of HACS. We found that a significantly increase of luciferase activity in the Link29-PAKKKR. These findings indicated that the enlarged stem-loop structures accelerated the nucleotide insertions into the cleavage site. To further confirm our hypothesis, we constructed another modified Link29-PAKSKR-NL and Link29-PAKKKR-NL plasmids, which were artificially designed to have consecutive adenines and to minimize the loop structures (Fig. 4c). As expected, compared to the enlarged loop structure of Link29-PAKKKR, the significantly lower luciferase expression was observed in the DF-1 cells when transfected with Link29-PAKKKR-NL; however, the difference of luciferase expression between Link29-PAKSKR and Link29-PAKSKR-NL was not significant (Fig. 4a; Supplementary Data 8). These findings suggested that the enlarged stem-loop magnitude was correlated with the efficiency of luciferase expression.

To verify the presence of nucleotide insertions in the different HACS of H9N2 viruses, we analyzed the linker RNA of different HACS in the DF-1 cells when transfected with different plasmids, including Link29-PARSSR, Link29-PAKSSR, Link29-PSRSSR, Link29-PAKSKR, Link29-PAKKKR, and Link29-PAASDR plasmids. Using Sanger sequencing, the single-nucleotide and double-nucleotide insertions or double-nucleotide deletion occurred in the synthetize of mRNA or vRNA in the enlarged stem-loop RNA secondary structure of HACS in H9N2 viruses, while no insertions were observed in the linker RNA of other HACS motif of H9N2 viruses when transfected with other plasmids (Supplementary Fig. 10), indicating that the enlarged stem-loop structure accelerated frequency of the nucleotide insertion.

### The enlarged RNA secondary stem-loop structure of HACS do not influence viral replication in H9N2 virus

**Fig. 5:**
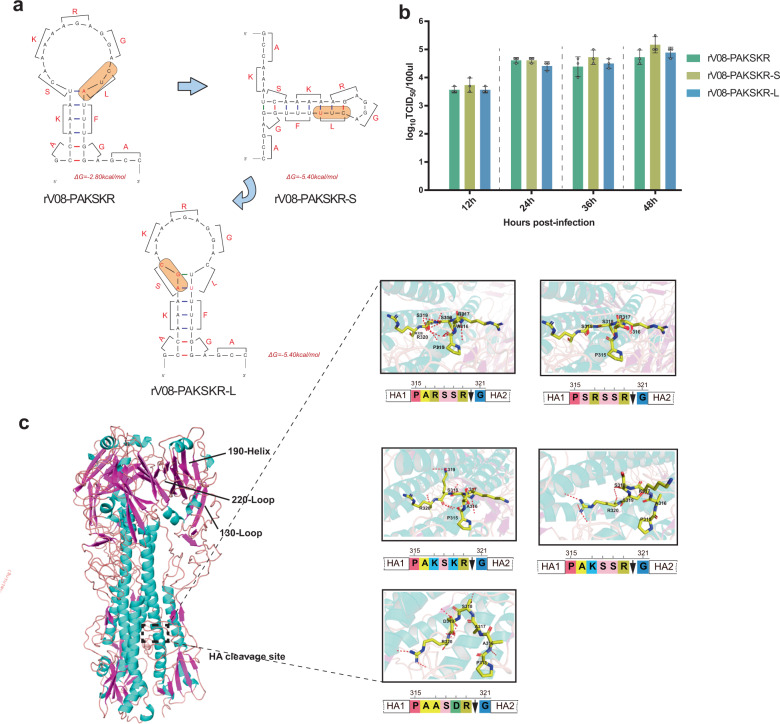
The structures of the tribasic HA cleavage sites and growth property of tribasic H9N2 viruses. **a** The predicted RNA secondary structures of tribasic HA cleavage sites and amino acids corresponding to each codon are shown. The Quickfold program (http://unafold.rna.albany.edu/?qDINAMelt/Quickfold) and RNAfold program from the ViennaRNA Web Services (http://rna.tbi.univie.ac.at/) and the ViennaRNA package were used to predict RNA sequences of the cleavage site regions of the HA genes. **b** Growth curves after inoculation of each virus at a multiplicity of infection of 0.001 into MDCK cell. Each point on the curve is the mean ± standard deviation from three independent experiments. **c** Three-dimensional (3D) structural analysis of amino acids at the HA cleavage site. A side view of the HA trimer structure of influenza A virus based on the uncleaved H3 monomer (protein Data Bank ID 4BSE) as a template. The corresponding amino acids to a 3D structure of the HA protein containing different cleavage motifs were mapped using MacPymol (http://www.pymol.org/).

To assess the correlation between the viral replication and the enlarged stem-loop structures of HACS, we constructed the synonymous mutation of the HACS in H9N2 virus, designating rV08-PAKSKR-S, which could minimize the stem-loop structures of HACS (Fig. 5a). The replicative ability of the rV08-PAKSKR-S was not significant when compared with that of rV08-PAKSKR. Subsequently, we restored the size of stem-loop structure of HACS and found that the replicative ability of the H9N2 viruses did not increase (Fig. 5b; Supplementary Data 9), indicating that viral replication was not responsible for the enlarged RNA secondary stem-loop structure in H9N2 viruses. We then analyzed the three-dimensional (3D) structural basis of the different HACS in H9N2 viruses. The structure of HACS showed that, with the exposed conformation, the cleavage site loop with K at residue 319 was more exposed to the glycoprotein surface, and thus, the potential proteases could more easily access the cleavage site. However, when the residue at P2 and P4 were both non-basic amino acids, the residues potentially blocked the surface and were not exposed in the same way, resulting in the poor potential protease access (Fig. 5c). These findings indicated that the viral replication was not responsible for the enlarged RNA secondary stem-loop structure of the tribasic H9N2 virus, and however, possibly due to the 3D amino acids structure of the HA protein.

### Using the predicted mutation plots to trace the evolution of HACS in H9N2 viruses

**Fig. 6:**
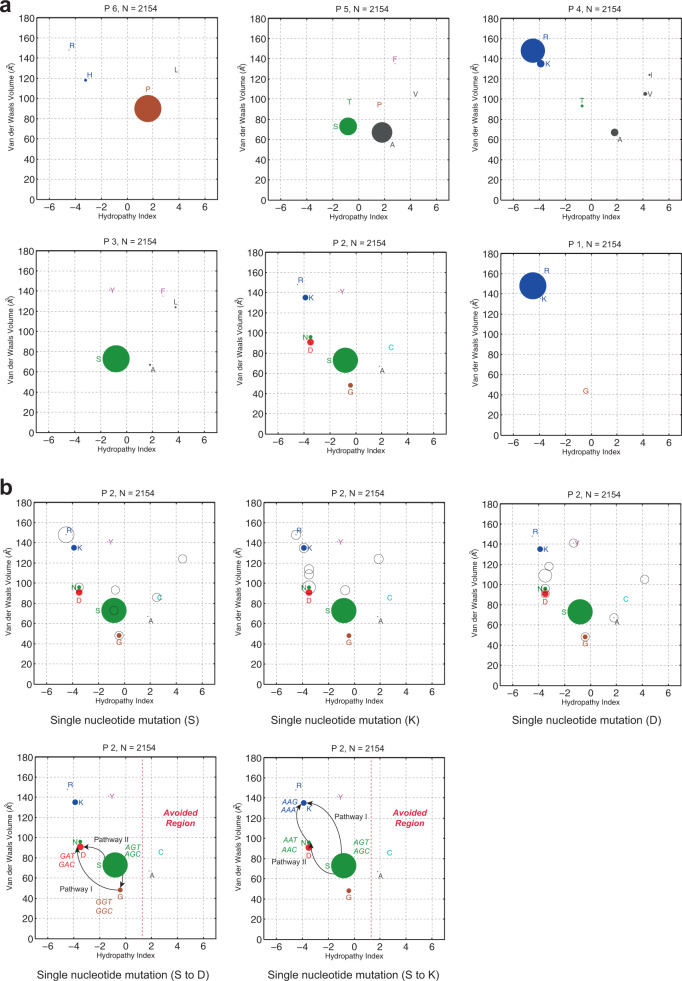
Scatter plot of the HA proteolytic cleavage site. **a** Scatter plots of each AA residue at the HACS of 1550 HA sequences. The *X*-axis is HPI (unitless), and the *Y*-axis is *Y*-axis is VdWV (Å^3^). **b** Scatter plots for P2 position residues. Potential mutation pathways are shown by an arrow. An “Avoided Region” was marked to show the biochemical properties that appear to be unfavorable for the P2 position mutation.

A bioinformatics tool described by Lee DW^29^, AAScatterPlot, was used to represent the diversity of amino acid residues at the HA proteolytic cleavage site from P6 to P1. The scatter plots showed the diversity and rare residues at each position more clearly than the WebLogo. The size and color of the dots corresponded to the frequency of occurrence and the unique properties of the residue (green = polar neutral, blue = polar positive, red = polar negative, brown = proline or glycine, teal =  bonding cysteine, and gray = nonpolar aliphatic). A random point mutation of a residue in a codon was indicated with a hollow circle overlaying the scatter plots. In our study, we found that the P6 and P1 positions were stable as compared to other residues during the viral evolution. However, substitutions with hydrophobic residues were observed in the P4 and P3 (Fig. 6a). The P5 and P2 seemed to mutate in a defined region, and P2 seemed to prefer hydrophilic residues during 2010 to 2019 (Supplementary Fig. 11). We then indicated an ‘Avoided Region’ to show the biochemical properties, which was unfavorable for the P2 mutation (Fig. 6b). AAScatterPlot was also used to predict the restricted evolutionary paths of the HA proteolytic cleavage site. According to the tool, we found that there would be two major paths for the P2 position to mutate between residues G and N, including S⇄G⇆D or S⇄N⇆K (Fig. 6b), which supported that the transition between P2 D and K would most likely involve a P2 G and N intermediate, respectively. It is noteworthy that the P2 N was increasing in the G1 and Y280 lineage H9N2 viruses in recent years (Supplementary Fig. 11), which indicated that the possible path for the P2 position from N to K. These findings suggested that the P2 residue was experiencing a selection pressure, and the tendency of the HACS evolving from dibasic to tribasic was observed in H9N2 viruses.

## Discussion

The H9N2 virus was one of the major subtype of influenza viruses circulating in poultry worldwide that causes significant economic losses to the poultry industries and threatens human health^5,7^. Because influenza A(H9N2) viruses were low pathogenic to birds and had only sporadically infected humans with mild or asymptomatic symptom, their eradication had not been a priority for zoonosis control agencies in many countries. This neglect caused them to continue to share genetic materials with other HP influenza virus of public health significance. Cleavage activation of HA by host protease is a crucial step in the life cycle of influenza virus and ultimately influence the virulence, transmissibility between hosts and adaption to new species^20,30–32^. The minimum sequence requirement of H9N2 viruses is R/K-X-X-R, which consisted of two paired arginine/lysine residues at the P1 and P4 position. However, we found that the HACS of H9N2 virus showed the avoidance of hydrophobic residues at the P2 and P5 positions, and the P2 position seems tolerant of large variations in biochemical properties, especially S337K substitution. Interestingly, our findings showed the mutation pathways that a virus can take to transition between a P2 S and K via a N (Fig. 6b), indicating that P2 residues are experiencing a selection pressure during virus evolution.

**Fig. 7:**
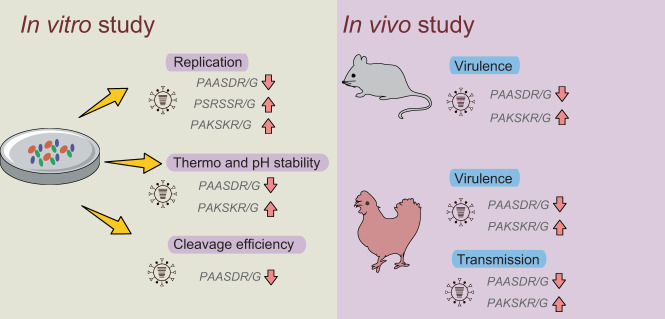
Graphic summary of H9N2 viruses containing the different cleavage site motifs in vivo and in vitro. The up arrow and down arrow indicate the increase and decease, respectively.

This study demonstrated that during serial passaging in chickens, the P2 position at HACS of the H9N2 virus was more polymorphic, and the amino acids N337K in the P2 position detected in the 6th passage^33^. The G1-lineage H9N2 viruses were adapted to birds and humans and induced more severe infections than the other representative lineage of H9N2 virus, and a basic amino acid in the P3 position of HACS formed an intermediate step towards a gain in pathogenicity^34^. In this study, we found that a sharp increase occurred after 2010 in the G1-lineage of H9N2 viruses with an identical HACS of the tribasic PAKSKR/G-motif from Bangladesh. Previous study showed that the tribasic H9N2 viruses can be activated by matriptase and furin^22,35^. It is noteworthy that all SARS-CoV-2 harbor tribasic cleavage sites, which are activated by ubiquitously expressed furin^36^. Furin and matriptase are mainly expressed in the kidneys and human lungs, indicating the potential to infect humans of H9N2 viruses. Our findings suggested that the predominant tribasic H9N2 virus increased the replicative ability, viral stability, and digestive virus spread in chickens (Fig. 7), which explains the selective evolution in the HACS that increase viral fitness in chickens. In addition, when the residues at P2 and P4 were both non-basic amino acids, the H9N2 virus dramatically reduced the stability, cleavage efficiency, pathogenicity and transmission in chickens (Fig. 7), indicating a decrease in viral fitness of monobasic H9N2 viruses. The North American-like and Y439-like H9N2 sublineages with P2 D had difficulty infecting chickens and pigs^37^, and this prototype virus did not become a dominant variant and cause human infection in China, which might be responsible for the lack of its transmission to birds.

The secondary structures of RNA molecules were important for the RNA editing mechanism^38^. The insertions encoding for multibasic cleavage site motifs in the HA gene of HP H5 and H7 subtype viruses occurred in the hairpin loops of the stem-loop structures flanking the cleavage site regions^39^. Compared to the RNA structures of cleavage sites among H5 and H7 subtype viruses, the stem-loop structure of H9N2 viruses tended to be smaller. However, interestingly, we found that the stem-loop structure of PAKSKR/G-motif was obviously larger than that of the other HACS, and it significantly enhanced the luciferase expression compared with the reporter plasmid containing the PARSSR/G-motif, which suggested that this enlarged stem-loop structure was important to accelerate the nucleotide insertion in HACS, indicating potential threats of acquiring the polybasic cleavage sites of H9N2 viruses. Although we observed that the tribasic cleavage site motif accelerated the nucleotide insertion in HACS, we cannot determine whether the tribasic H9 virus could evolve to become HP in chickens and posed increased threat to humans. Previous study indicated that the introduction of polybasic amino acid residues at the HACS of H9N2 virus alone could not acquire high pathogenicity in birds^40^, unless it undergoes a series of passages in chickens. HP is a generalized phenotype determined by a series of complex genotype changes in influenza virus, which could arise from different combinations of adaptive mutations and reassortment. In addition, disrupt of the HACS structure in H5 subtype mutant viruses generated by reverse genetics did not reveal any effect on viral replication^41^. Consistent with their findings, we found that enlarged RNA secondary stem-loop structure in H9N2 viruses could not increase the viral replication, indicating that no direct correlation between conservation of the RNA structure of influenza virus and their functional importance.

Due to the controversy of the gain-of-function study, we did not passage the tribasic or polybasic H9N2 viruses in chickens to explore the pathogenicity determinant of HP H9N2 viruses. However, it is noteworthy that the H9N2 viruses bearing the tribasic or polybasic cleavage sites with the internal genes of H5N1 viruses obviously enhanced the pathogenicity in chickens. Bangladesh and Egypt had been identified as a “hotspot” of H9N2 and H5N1 evolution in multiple avian species^25,42^. Although it remains speculative, the increasing tribasic H9N2 viruses suggests that a substantial potential to generate novel reassortants with greater fitness in avian and mammalian species, and in turn, evolving into the HP phenotype. The Middle East, south Asia, and Africa in particular have an important role in the global dissemination of H9N2 virus, since these countries here lie along major migratory bird pathways–the central Asian, east Africa-west Asian, and Black Sea-Mediterranean routes. Considering these concern, it should be noted that the transmission of the H9N2 virus accelerates the spread and reassortment of novel human-infecting influenza viruses. Our findings initially offer insights into the evolutionary dynamics of HACS of H9N2 viruses and risk assessment regarding which predominant cleavage site motifs may pose the great threat for zoonotic and pandemic emergence in birds and humans.

## Methods

### Ethics statement and biosafety

All experiments with reverse H9N2 avian influenza viruses (AIVs) were conducted in an animal biosafety level 3 laboratory and animal facilities at South China Agricultural University (SCAU) (CNAS BL0011) in accordance with protocols. All animals involved in the experiments were reviewed and approved by the Institution Animal Care and Use Committee at SCAU and treated in accordance with the guidelines (2017A002).

### Data collection

All of the human-infecting influenza A (H9N2) cases data were obtained from the World Health Organization (WHO) (http://www.who.int/en/), the Food and Agriculture Organization of the United Nations (EMPRES-I data sets) (http://empres-i.fao.org/eipws3g/#h=1), and the National Health Commission of the People’s Republic of China (http://en.nhfpc.gov.cn/). We performed a systematic search using PubMed, Internet-based, the Weekly Epidemiological Record (WER) of WHO for serological evidence of human infection. The global distributions of influenza A (H9N2) viruses in birds and humans were available from GenBank (http://ncbi.nlm.nih.gov/genbank/) and GISAID (https://www.gisaid.org) and evaluated using GraphPad Prism 7 (https://www.graphpad.com/) and ArcGIS 10.4 for Desktop (http://www.esri.com/software/arcgis/arcgis-for-desktop/).

### Virus isolation and cells

The virus used, the H9N2 virus A/chicken/Guangdong/V/2008 (rV08-PARSSR) (PB2, EPI1383190; PB1, EPI1383191; PA, EPI1383192; HA, EPI1383193; NP, EPI1383194; NA, EPI1383195; M, EPI1383196; NS, EPI1383197) was isolated from ostensibly healthy chickens in a poultry farm in Guangdong Province of China in 2008. Each segment used in this study is available in the Global Initiative on Sharing All Influenza Data (GISAID) database (http://www.gisaid.org/). The H9N2 virus was propagated in the allantoic cavities of 10-day-old specific-pathogen-free (SPF) embryonated chicken eggs at 37 °C for 60 h and then were stored viruses at −80 °C until use. All cells used, including human embryonic kidney (HEK293T) cells, MDCK cells, A549 cells, CEFs cells, Vero cells, and DF-1 cells were provided by the National Avian Influenza Para-Reference Laboratory (Guangzhou) at South China Agricultural University. HEK293T, MDCK, A549, and Vero cells were grown in Dulbecco’s modified Eagle’s medium supplemented with 10% calf serum, 100U/ml penicillin, and 0.1 mg/ml streptomycin and incubated at 37 °C in a 5% CO_2_ incubator. CEFs and DF-1 cells were grown in Dulbecco’s modified Eagle’s medium supplemented with 10% calf serum, 100U/ml penicillin, and 0.1 mg/ml streptomycin and incubated at 39 °C in a 5% CO_2_ incubator.

### Phylogenetic analysis and generation of the biophysical scatter plots

All of the available genomic sequences with the complete coding regions of influenza A (H9N2) viruses were downloaded from GenBank (http://ncbi.nlm.nih.gov/genbank/) and GISAID (https://www.gisaid.org). The genomic sequence data set (sequence alignments are available on request) was then created. All sequences were aligned using the MAFFT (version 7.149) program (https://mafft.cbrc.jp/alignment/software/). Maximum likelihood (ML) phylogenies for the codon alignment of the genomic gene segments were estimated using the GTRGAMMA nucleotide substitution model in the RAxML (version 8.2) program. Node support was determined by nonparametric bootstrapping with 1000 replicates. The phylogenetic tree was visualized with the FigTree (version 1.4.3) program (http://tree.bio.ed.ac.uk/software/figtree/). The AAscaterPlot was coded in MATLAB (MathWorks), and the source codes and installation file were provided at http://github.com/WhittakerLab/AAScaterPlot.

### Plasmid construction and reverse genetics

Eight gene segments from the A/chicken/Guangdong/V/2008 (rV08-PARSSR) strain were cloned into the Hoffmann’s bidirectional transcription vector pHW2000 plasmid system^43^, as described previously. The parental and HA mutant constructs (rV08-PAKSSR, rV08-PSRSSR, rV08-PAKSKR, and rV08-PAASDR) were produced by site-directed mutagenesis PCR and then sequenced. The recombinant H9N2 viruses were designated rV08-PARSSR, rV08-PSRSSR, rV08-PAKSSR, rV08-PAKSKR, and rV08-PAASDR, respectively. The five H9N2 viruses were generated using reverse genetics. Briefly, HEK293T cell monolayers in 6-well plates were transfected at 80-90% confluency with 4 μg of the eight plasmids (500 ng of each plasmid) by using Lipofectamine 2000 (Invitrogen) according to the manufacturer’s instructions. DNA and transfection reagent were mixed and incubated at room temperature for 5 min, and added to the cells. Four hours later, the mixture was replaced with Opti-MEM (GIBCO) containing 0.2% bovine serum albumin and 1 μg/ml trypsin. After 48 h, the supernatant was harvested and injected into SPF embryonated eggs for virus propagation. Viruses were titrated in embryonated eggs using hemagglutination assays, as recommended by the WHO manual on influenza diagnosis and surveillance. All mutant viruses were confirmed by RT-PCR and sequencing.

### Animal experiments

For the chicken experiment, groups of ten 5-week-old SPF chickens (Guangdong Dahuanong Animal Health Products Co., Ltd., Guangdong Province, China) were inoculated intranasally with 10^6^ EID_50_/200 μl of the five H9N2 viruses. Chickens were observed for clinical symptoms for 14 days, and serum samples were collected for analysis of virus-specific antibodies. Ten chickens were introduced into the isolators with inoculated chickens 24 h after direct inoculation to assess transmission. Cloacal and throat swab specimens were collected at 3, 5, 7, 9, and 11 dpi from the infected and naïve chickens. Three chickens in each group were dissected to test for virus replication in the organs, including the lung, kidney, duodenum, and brain. Tissue and swab samples were collected for virus titration by an EID_50_ assay. For the mouse experiments, groups of 4-week-old female BALB/c mice, obtained from the Vital River Company in Beijing, were anesthetized with isoflurane and inoculated intranasally with 10^6^ EID_50_/50 μl viruses. To test for virus replication in the organs, three mice in each group were euthanized at 4 dpi. Tissue samples including brain and lung tissue samples from each euthanized mouse were collected for virus titration by an EID_50_ assay.

### Thermal stability and syncytia formation assay to evaluate the threshold pH of HA activation

The thermal stability of the HA protein was measured by determining the loss of the virus titer after incubating the viruses at 50 °C temperature for 1–5 h. The titers of heat-treated H9N2 viruses were determined by TCID_50_ assay in MDCK cells. Vero cells grown in 24-well plates were inoculated with five H9N2 viruses containing different HACS motifs at an MOI of 1 for 16 h. Vero cell cultures were then treated with 10 μg/ml of TPCK-trypsin (Sigma) at 37 °C for 15 min. To induce syncytia formation, cells were incubated with fusion buffer with the pH adjusted to 5.0, 5.2, 5.4, 5.6, and 5.8 for 5 min at 37 °C. At 3 h post fusion induction, Vero cells were fixed and stained with Giemsa stain (Solarbio Life Science, Beijing, China). Images were taken on a cell imaging system (Leica DM14800B, Germany).

### Western blotting

CEF and A549 cells were inoculated with the H9N2 viruses at an MOI of 0.01 in the presence of either 1 μg/ml TPCK-trypsin for 24 h in minimal essential medium containing 0.2% bovine serum albumin (BSA) (Dingguo, Beijing, China). Proteins from the cell cultures were separated on 8% sodium dodecyl sulfate-polyacrylamide gel electrophoresis (SDS-PAGE) gels and then electrotransferred onto nitrocellulose membranes. Polyclonal rabbit anti-HA antibody from A/Hong Kong/1073/99 H9N2 expressed in baculovirus (1:2000 dilution for 2 h at room temperature; Sino Biological Inc., Beijing, China) was used to detect HA. Goat anti-mouse IgG conjugated with horseradish peroxidase (1:10,000 dilution for 1 h at room temperature; Dingguo, Beijing, China) was used as a secondary antibody, followed by chemiluminescence detection (LI-COR Odyssey). Grayscale analysis of individual bands was determined by using ImageJ, and relative cutting efficiency was calculated according to the equation HA1/(HA0 + HA1).

### Virus growth kinetics

Confluent MDCK cells were infected at a multiplicity of infection (MOI) of 0.001 TCID_50_/cell for 1 h at 37 °C, and CEF cells were infected at an MOI of 0.001 TCID_50_/cell for 1 h at 37 °C. After 1 h incubation, the cells were washed twice, and then incubated with DMEM containing 0.2% BSA (Dingguo, Beijing, China) and TPCK trypsin (1 μg/ml) at 37 °C with 5% CO_2_. Culture supernatants were collected at the indicated time points and stored at −80 °C until use. The MDCK cells infected with rV08-PAKSKR appeared cytopathic effect and began to fall off slightly after 24 hpi. Hence, the mixture of culture supernatants and cell cultures were collected at the indicated time points, and viral titers were determined by the TCID_50_ assay in MDCK cells.

### Reporter assay

In brief, we used the modified luciferase reporter plasmid, containing chicken RNA polymerase I promoter, RNA polymerase I terminator, rV08-PARSSR HA segment-derived noncoding region (NCR), and the firefly luciferase gene (Supplementary Fig. S4). The reporter plasmid was constructed by inserting 29-polynucleotide and 30-polynucleotide linkers whose sequences were derived from those encoding the HA cleavage site of the rV08-PARSSR strains, between a start codon and the remaining ORF of the firefly luciferase gene. The construct was flanked by the rV08-PARSSR HA-NCR at both the 5’ and 3’ ends. The RNP complexes, consisting of PA, PB1, PB2, and NP (200 ng each) plasmids of rV08-PARSSR virus, were mixed with a luciferase reporter plasmid (200 ng) and a thymidine kinase promoter-Renilla luciferase reporter plasmid (pRL_TK) construct (20 ng), then co-transfected into DF-1 cells in a 12-well plate with Lipofectamine 3000 (Invitrogen, Carlsbad, CA), and incubated at 39 °C for 24 h. Luciferase production was assayed using the dual-luciferase reporter assay system (Promega) according to the manufacturer’s instructions. Firefly luciferase activities were standardized to the transfection control *Renilla* luciferase activities (i.e., firefly luciferase activities were divided by *Renilla* luciferase activities).

To verify the presence of nucleotide insertions in the different HACS of H9N2 viruses, RNA was extracted from the transfected DF-1 cells with the RNeasy Mini Kit (Qiagen) as directed by the manufacturer. Then, RNA was treated with DNAase (Omega) to eliminate the influence of plasmids. Two-step RT-PCR was conducted with primers (F: 5’-TGTAGGAGATCTTCTAGAAAGATGTTAA-3’; R: 5’-ACTGCATACGACGATTCTGTGATT-3’). PCR products were purified with a QIAamp Gel extraction kit (Qiagen) and cloned into the *PJET* vector. Recombinant plasmids were maintained under nonselective conditions in Escherichia coli and then ten cloned plasmids of Link29-PARSSR, Link29-PSRSSR, Link29-PAKSSR, Link29-PAKSKR, Link29-PAASDR, and Link29-PAKKKR were sequenced with an ABI 3730 DNA Analyzer (Applied Biosystems), respectively.

### Prediction of the RNA secondary structure

The Mfold program (http://unafold.rna.albany.edu/?q=mfold), Quickfold program (http://unafold.rna.albany.edu/?qDINAMelt/Quickfold) and RNAfold program from the ViennaRNA Web Services (http://rna.tbi.univie.ac.at/) and the ViennaRNA package were used to predict RNA sequences of the cleavage site regions of the HA genes^44^. The HACS sequences of low pathogenic H5, H7, and H9 precursors were all avian species (chickens, ducks, and wild birds) available in the GISAID database (http://www.gisaid.org/). Identical HACS sequences were removed. The representative HA sequences of A/chicken/Bangladesh/29588/2016 (H9N2), A/chicken/Zhejiang/221/2016 (H9N2), A/chicken/Shenzhen/9/1997 (H9N2), A/chicken/Egypt/S4454B/2010 (H9N2), and A/wild waterfowl/Korea/7/2010 (H9N2), A/wild_caterfowl/Dongting/C2032/2011 (H9N2) isolates available in the GISAID’s EpiFlu Database (http://www.gisaid.org/) were used for the structural analysis.

### Structure-based mapping analysis

We predicted the HA monomer structure using the SWISS-Model website (https://swissmodel.expasy.org/), employing the uncleaved H3 monomer (protein Data Bank ID 4BSE) as a template. The corresponding amino acid to a three-dimensional (3D) amino acids structure of the HA protein were mapped using MacPymol (http://www.pymol.org/).

### Statistical analysis and reproducibility

Data are presented as the mean ± SD and analyzed using GrapPad Prism 5.0. The independent-samples *t* test was applied for analysis, and *p* values less than 0.05 were considered significant and are represented as *p < 0.05, **p < 0.01, ***p < 0.001, ****p < 0.0001. For all statistically significant data from at least two biological repeats performed on separate days was used. The exact number of replicates are presented in individual figure legends. Any differences in statistical significance are indicated.

### Reporting summary

Further information on research design is available in the Nature Research Reporting Summary linked to this article.

## Supplementary information

Peer Review File

Supplementary Information

Description of Additional Supplementary Files

Supplementary Data 1

Supplementary Data 2

Supplementary Data 3

Supplementary Data 4

Supplementary Data 5

Supplementary Data 6

Supplementary Data 7

Supplementary Data 8

Supplementary Data 9

Reporting Summary

## Data Availability

Source data underlying the graphs and charts in the main figures are available in Supplementary Data 2–9. Full blots are shown in Supplementary Information. All other data that support the findings of this study are available from the corresponding author (W.Q.) upon reasonable request.
