# Supplementary material for: A risk marker of tribasic hemagglutinin cleavage site in influenza A (H9N2) virus
Source: Commun Biol. 2021 Jan 15;4:71. doi: 10.1038/s42003-020-01589-7 (PMC7811019; doi:10.1038/s42003-020-01589-7)
Supplement: Supplementary file 2 — Supplementary Information [file 42003_2020_1589_MOESM2_ESM.pdf]

A risk marker of tribasic hemagglutinin cleavage site in influenza A (H9N2) virus

Jiahao Zhang, Kaixiong Ma, Bo Li, Yiqun Chen, Ziwen Qiu, Jinchao Xing, Jinyu Huang, Chen Hu, Yifan Huang, Huanan Li, Dingxiang Liu, Ming Liao, and Wenbao Qi

Supplementary Information

Supplementary Figure 1

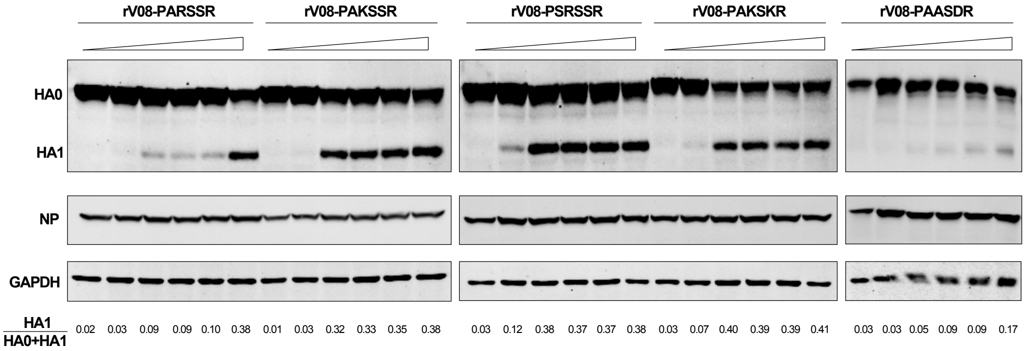

Supplementary Figure 1. Western blots of CEF cells inoculated with five influenza A(H9N2) virus at an MOI of 0.01 in the presence of 0, 0.2, 0.4, 0.6, 0.8, and 1.0 μg/ml TPCK-trypsin for 24 h.

Supplementary Figure 2

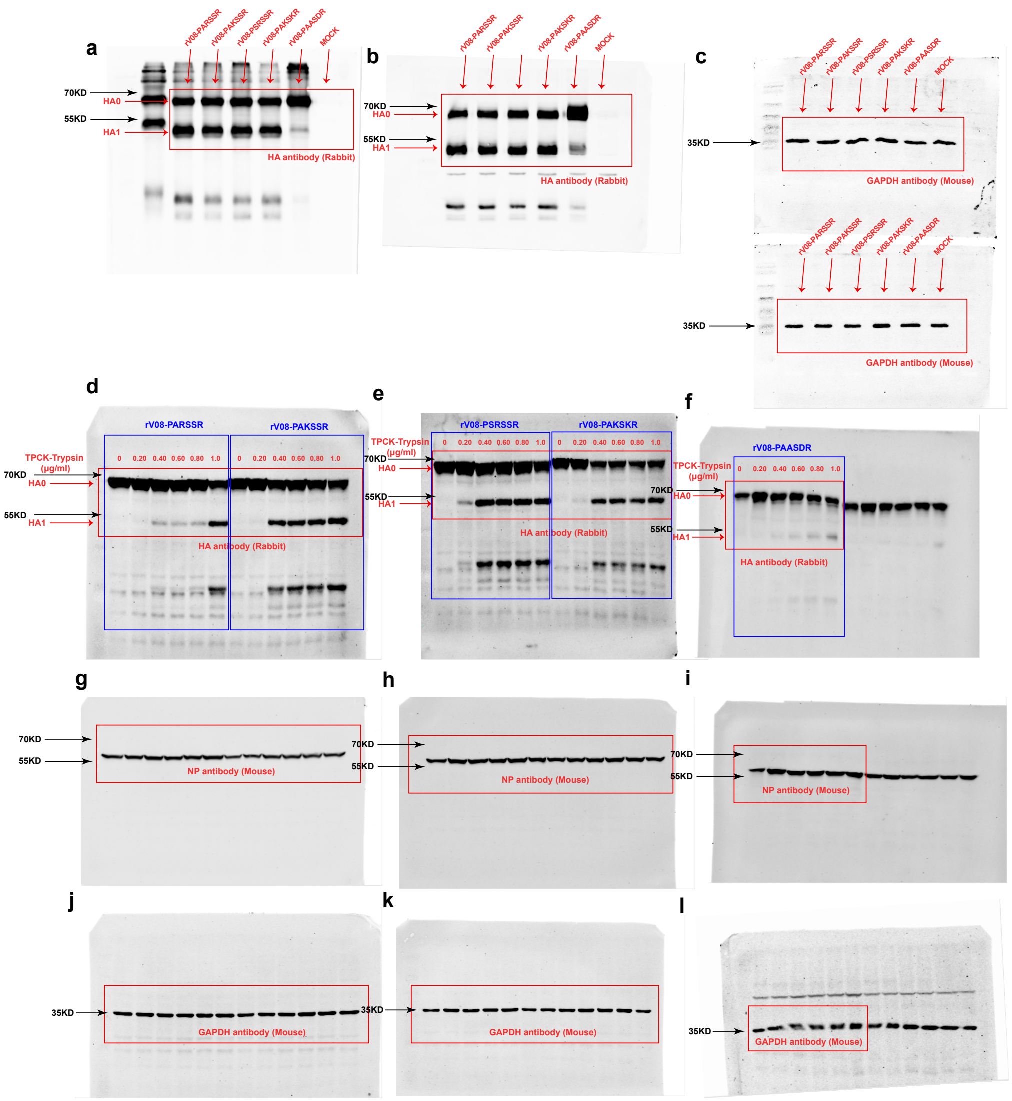

Supplementary Figure 2. The original picture of Western blots used in this study.

The Supplementary Figure 2a-c correspond to the Figure 3d. The Supplementary Figure 2d-l correspond to the Supplementary Figure 1.

Supplementary Figure 3

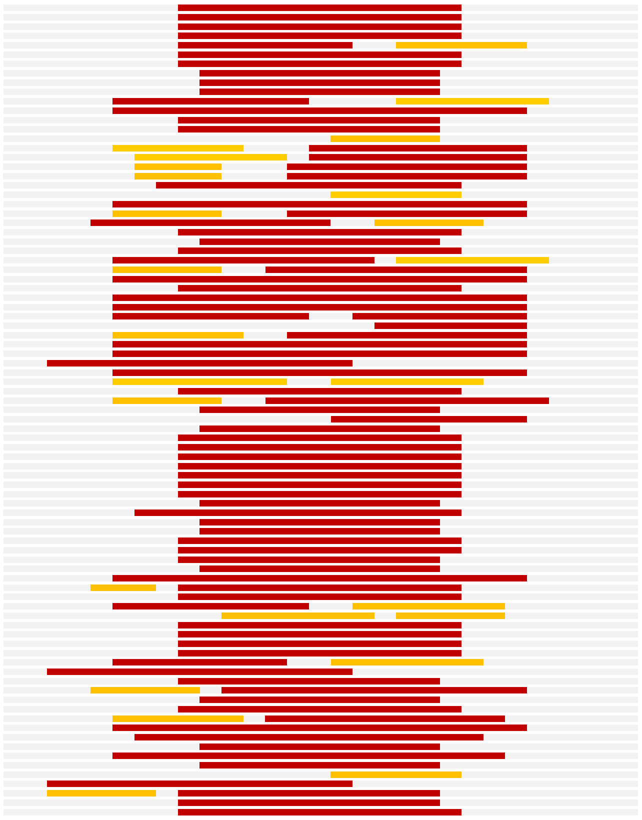

Supplementary Figure 3. The stem-loop structure in the different HACS of low pathogenic H5 subtype influenza viruses. The horizontal bars represent the HACS region, and the red and orange lines indicate the stem-loop structures consisting of more than 8 nucleotides that are fully included the codons for arginine and glycine in the HACS and the others, respectively.

Supplementary Figure 4

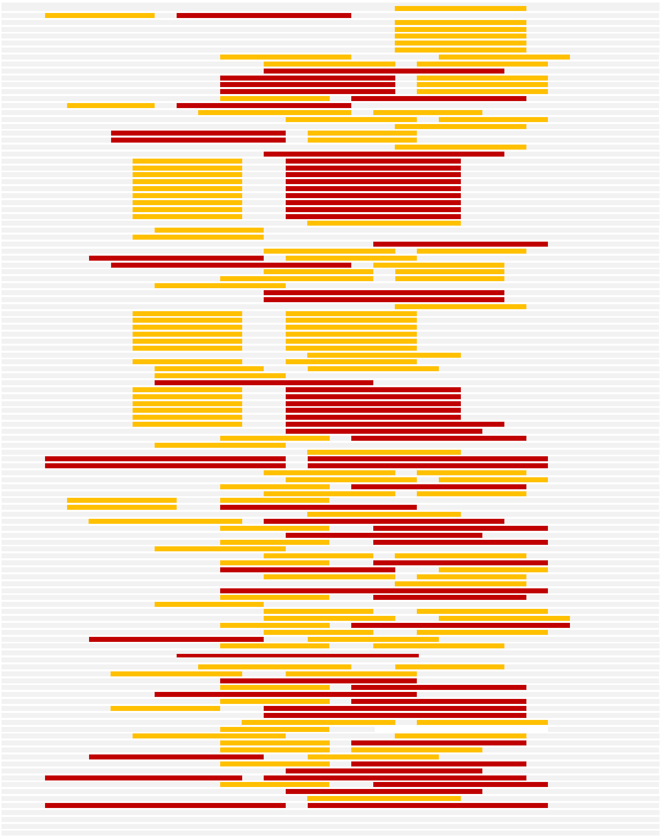

Supplementary Figure 4. The stem-loop structure in the different HACS of low pathogenic H7 subtype influenza viruses. The horizontal bars represent the HACS region, and the red and orange lines indicate the stem-loop structures consisting of more than 8 nucleotides that are fully included the codons for arginine and glycine in the HACS and the others, respectively.

Supplementary Figure 5

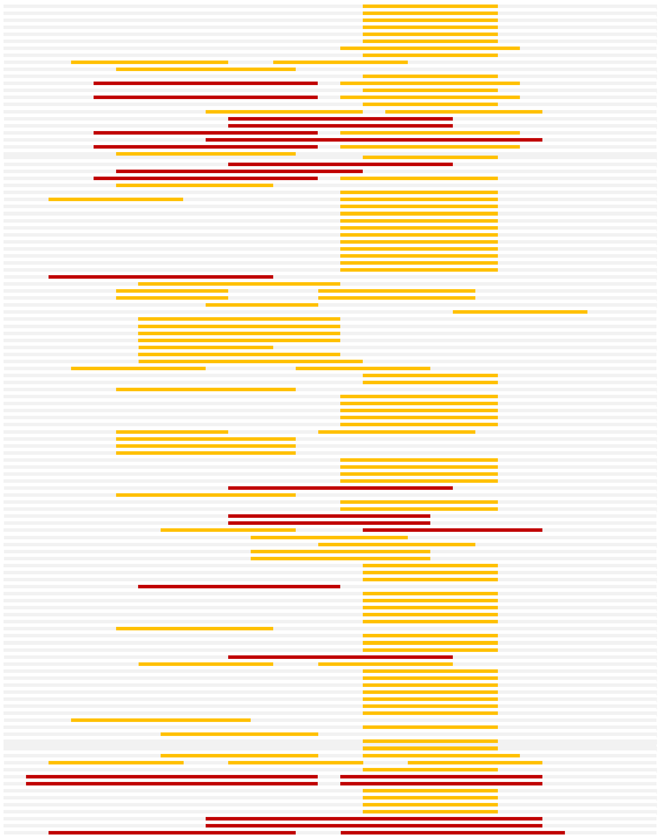

Supplementary Figure 5. The stem-loop structure in the different HACS of low pathogenic H9 subtype influenza viruses. The horizontal bars represent the HACS region, and the red and orange lines indicate the stem-loop structures consisting of more than 8 nucleotides that are fully included the codons for arginine and glycine in the HACS and the others, respectively.

Supplementary Figure 6

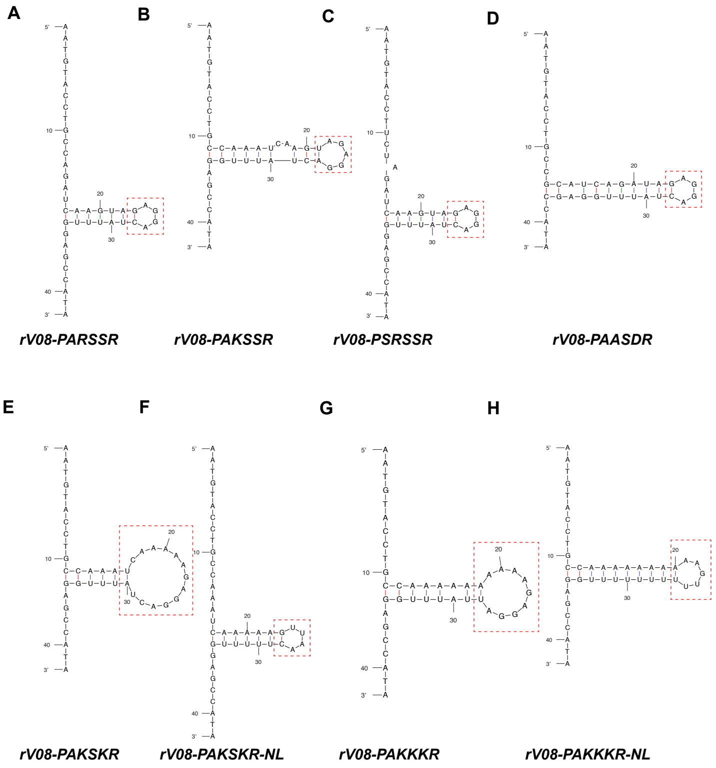

Supplementary Figure 6. Quickfold-predicted RNA secondary structures in the different HACS linker (40 nt) of H9N2 viruses. The predicted RNA secondary stuctures of rV08-PARSSR **(A)**, rV08-PAKSSR **(B)**, rV08-PSRSSR **(C)**, rV08-PAASDR **(D)**, rV08-PAKSKR **(E)**, rV08-PAKSKR-NL **(F)**, rV08-PAKKKR **(G)**, and rV08-PAKKKR-NL **(H)** are shown.

Supplementary Figure 7

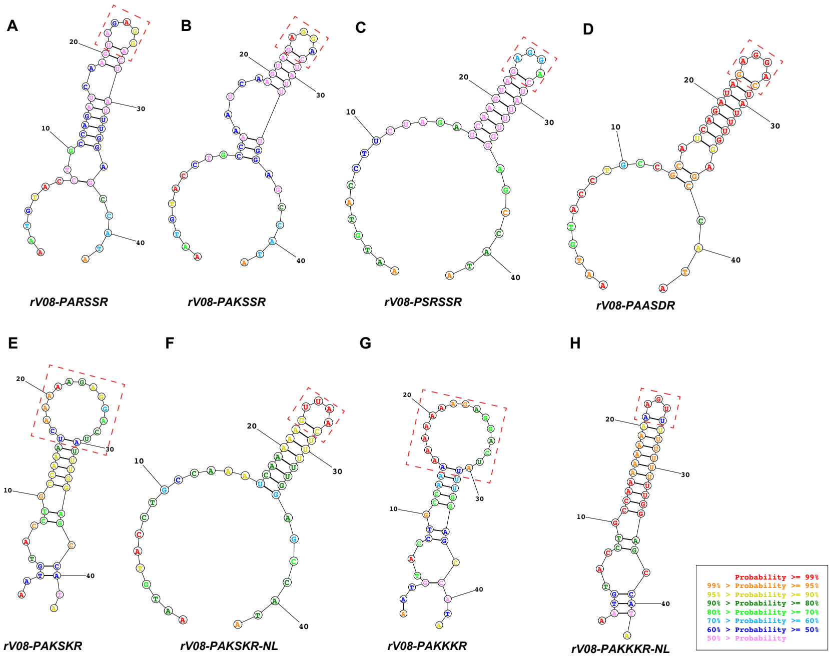

Supplementary Figure 7. Mfold-predicted RNA secondary structures in the different HACS linker (40 nt) of H9N2 viruses. The predicted RNA secondary stuctures of rV08-PARSSR **(A)**, rV08-PAKSSR **(B)**, rV08-PSRSSR **(C)**, rV08-PAASDR **(D)**, rV08-PAKSKR **(E)**, rV08-PAKSKR-NL **(F)**, rV08-PAKKKR **(G)**, and rV08-PAKKKR-NL **(H)** are shown.

Supplementary Figure 8

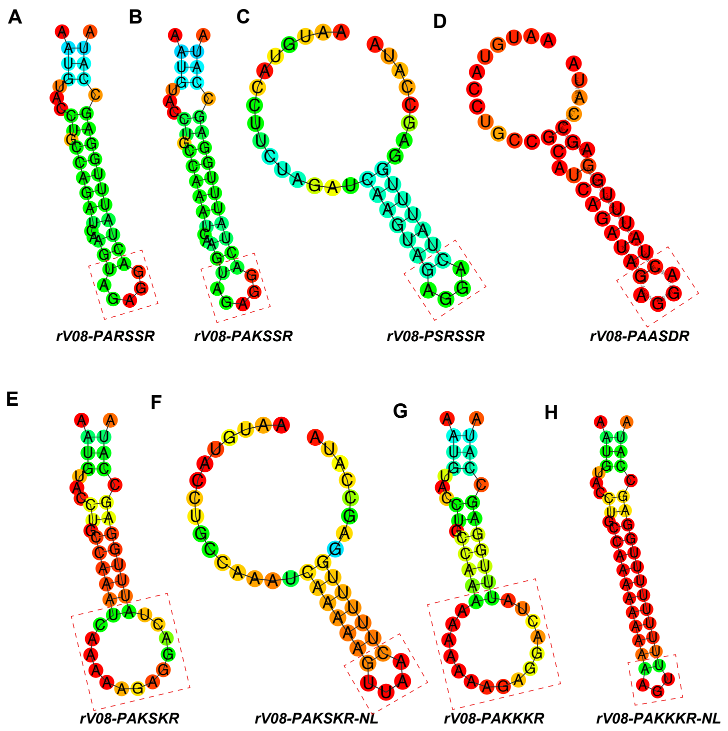

Supplementary Figure 8. RNAfold-predicted RNA secondary structures in the different HACS linker (40 nt) of H9N2 viruses. The predicted RNA secondary stuctures of rV08-PARSSR **(A)**, rV08-PAKSSR **(B)**, rV08-PSRSSR **(C)**, rV08-PAASDR **(D)**, rV08-PAKSKR **(E)**, rV08-PAKSKR-NL **(F)**, rV08-PAKKKR **(G)**, and rV08-PAKKKR-NL **(H)** are shown.

Supplementary Figure 9

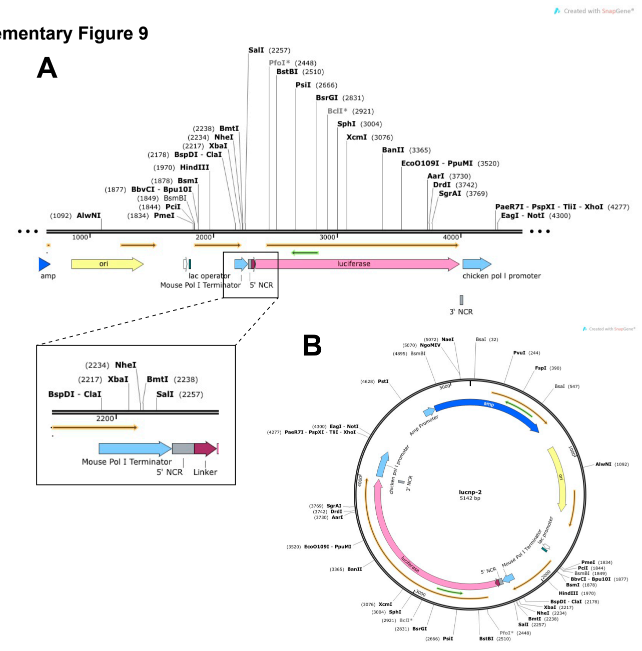

Supplementary Figure 9. A map of modified luciferase reporter plasmid containing chicken RNA polymerase I promoter, RNA polymerase I terminator, rV08-PARSSR HA segment-derived noncoding region (NCR), and the firefly luciferase gene.

Supplementary Figure 10

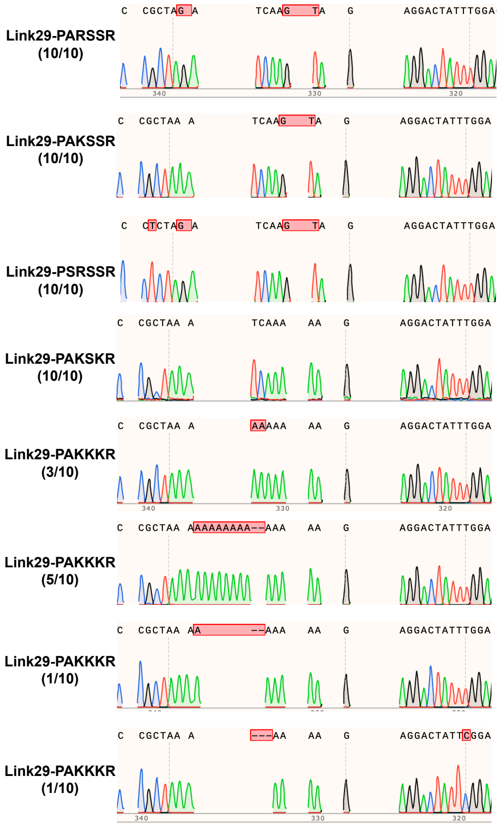

Supplementary Figure 10. The sequenced HACS linkers of H9N2 viruses bearing the different HACS motifs in H9N2 viruses. RNA was extracted from the transfected DF-1 cells with the RNeasy Mini Kit (Qiagen) as directed by the manufacturer. Then, RNA was treated with DNAase (Omega) to eliminate the influence of plasmids. Two-step RT-PCR was conducted with primers (F: 5’ TGTAGGAGATCTTCTAGAAAGATGTTAA-3’; R: 5’-ACTGCATACGACGATTCTGTGATT-3’). PCR products were purified with a QIAamp Gel extraction kit (Qiagen) and cloned into the *PJET* vector. Recombinant plasmids were maintained under nonselective conditions in Escherichia coli, and the ten cloned plasmids of each groups were sequenced.

Supplementary Figure 11

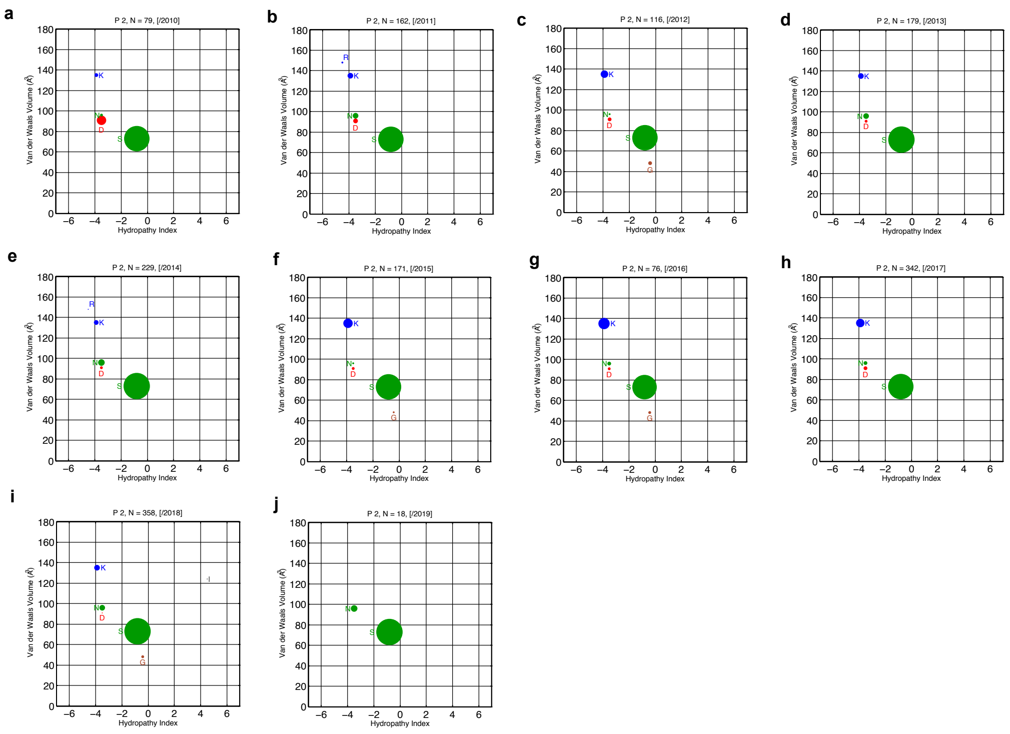

Supplementary Figure 11. Web logo and scatter plot representations of the HACS. Redrawn scatter plots for P2 position for each year from 2010 **(a)**, 2011 **(b)**, 2012 **(c)**, 2013 **(d)**, 2014 **(e)**, 2015 **(f)**, 2016 **(g),** 2017 **(h)**, 2018 **(i)**, 2019 **(j)**.
