# Supplementary material for: A risk marker of tribasic hemagglutinin cleavage site in influenza A (H9N2) virus
Source: Commun Biol. 2021 Jan 15;4:71. doi: 10.1038/s42003-020-01589-7 (PMC7811019; doi:10.1038/s42003-020-01589-7)
Supplement: Supplementary file 3 — Description of Additional Supplementary Files [file 42003_2020_1589_MOESM3_ESM.pdf]

Description of Additional Supplementary Files

File Name: Supplementary Data 1

Description: Primers used in this study.

File Name: Supplementary Data 2

Description: The raw data of growth curves after inoculation of each virus at a multiplicity of infection of 0.001 into MDCK cells.

File Name: Supplementary Data 3

Description: The raw data of growth curves after inoculation of each virus at a multiplicity of infection of 0.001 into CEF cells.

File Name: Supplementary Data 4

Description: The raw data of thermo stability of the influenza A(H9N2) viruses. The titers of heat-treated recombination viruses were determined by $TCID_{50}$ assay in MDCK cells.

File Name: Supplementary Data 5

Description: The raw data of viral titers in the lung, kidney, duodenum, and brain of inoculated and exposed chickens. The raw data of viral titers in the lung and brain of infected mice.

File Name: Supplementary Data 6

Description: The infection rate in chicken cloacal and oropharyngeal swabs. Ten 5-week-old SPF chickens were inoculated with 10^6 EID_{50} of five H9N2 viruses in a volume of 200 μl. Cloacal and oropharyngeal swabs were collected on 3, 5, 7, 9, and 11 dpi from inoculated and exposed chickens.

File Name: Supplementary Data 7

Description: The raw data of antibody titers of inoculated and exposed chickens at 14 dpi.

File Name: Supplementary Data 8

Description: The raw data of luciferase activities were expressed relative to the Link30-PARSSR plasmid and compared among the reporter plasmids containing the different linkers.

File Name: Supplementary Data 9

Description: The raw data of growth curves after inoculation of each virus at a multiplicity of infection of 0.001 into MDCK cell in Fig. 5b.
