# Supplementary material for: A risk marker of tribasic hemagglutinin cleavage site in influenza A (H9N2) virus
Source: Commun Biol. 2021 Jan 15;4:71. doi: 10.1038/s42003-020-01589-7 (PMC7811019; doi:10.1038/s42003-020-01589-7)
Supplement: Supplementary file 13 — Reporting Summary [file 42003_2020_1589_MOESM13_ESM.pdf]

nature research

Corresponding author(s): Wenbao Qi; COMMSBIO-20-1666-A

Last updated by author(s): Oct 29, 2020

Reporting Summary

Nature Research wishes to improve the reproducibility of the work that we publish. This form provides structure for consistency and transparency in reporting. For further information on Nature Research policies, see our Editorial Policies and the Editorial Policy Checklist.

Statistics

For all statistical analyses, confirm that the following items are present in the figure legend, table legend, main text, or Methods section.

n/a	Confirmed	
☐	☒	The exact sample size (*n*) for each experimental group/condition, given as a discrete number and unit of measurement
☐	☒	A statement on whether measurements were taken from distinct samples or whether the same sample was measured repeatedly
☒	☐	The statistical test(s) used AND whether they are one- or two-sided<br>*Only common tests should be described solely by name; describe more complex techniques in the Methods section.*
☒	☐	A description of all covariates tested
☒	☐	A description of any assumptions or corrections, such as tests of normality and adjustment for multiple comparisons
☒	☐	A full description of the statistical parameters including central tendency (e.g. means) or other basic estimates (e.g. regression coefficient) AND variation (e.g. standard deviation) or associated estimates of uncertainty (e.g. confidence intervals)
☐	☒	For null hypothesis testing, the test statistic (e.g. *F*, *t*, *r*) with confidence intervals, effect sizes, degrees of freedom and *P* value noted<br>*Give P values as exact values whenever suitable.*
☒	☐	For Bayesian analysis, information on the choice of priors and Markov chain Monte Carlo settings
☒	☐	For hierarchical and complex designs, identification of the appropriate level for tests and full reporting of outcomes
☒	☐	Estimates of effect sizes (e.g. Cohen's *d*, Pearson's *r*), indicating how they were calculated

Our web collection on statistics for biologists contains articles on many of the points above.

Software and code

Policy information about availability of computer code

Data collection	MEGA (v7.0), MAFFT (v7.149), RAxML (v8.2), FigTree (v1.4.3), AAscaterPlot, SWISS-Model, MacPymol, ImageJ, GrapPad Prism (v5.0), ArcGIS (v10.4), Mfold, RNAfold, and Quickfold were used to data collection and analysis. Exact details are provided in the methods section.
Data analysis	MEGA (v7.0) and MAFFT (v7.149) were used to align the genomic sequences.<br>RAxML (v8.2) was used to conduct the phylogenic analysis.<br>The phylogenetic tree was visualized with the FigTree (v1.4.3).<br>AAscaterPlot was used to represent the diversity of amino acid residues at the HA proteolytic cleavage site, which was provided at http://github.com/WhittakerLab/AAScaterPlot.<br>SWISS-Model was used to predicted the HA monomer structure of the HA protein.<br>The corresponding amino acid to a three-dimensional (3D) amino acids structure of the HA protein were mapped using MacPymol.<br>Grayscale analysis of individual bands was determined by using ImageJ.<br>GraphPad Prism (v5.0) was used to represent data and for statistical analysis.<br>ArcGIS (v10.4) was used to conduct the global map.<br>Mfold, RNAfold, and Quickfold program was used to predict RNA sequences of the cleavage site regions of the HA genes.

For manuscripts utilizing custom algorithms or software that are central to the research but not yet described in published literature, software must be made available to editors and reviewers. We strongly encourage code deposition in a community repository (e.g. GitHub). See the Nature Research guidelines for submitting code & software for further information.

Data

Policy information about availability of data

All manuscripts must include a data availability statement. This statement should provide the following information, where applicable:

- Accession codes, unique identifiers, or web links for publicly available datasets
- A list of figures that have associated raw data
- A description of any restrictions on data availability

The plasmids and viruses are freely available from the authors. All source data underlying the graphs and charts in the main figures are available in the Supplementary Data section. The data that support the findings of this study are available from the corresponding author (W.Q.) upon reasonable request.

Field-specific reporting

Please select the one below that is the best fit for your research. If you are not sure, read the appropriate sections before making your selection.

☒ Life sciences ☐ Behavioural & social sciences ☐ Ecological, evolutionary & environmental sciences

For a reference copy of the document with all sections, see nature.com/documents/nr-reporting-summary-flat.pdf

Life sciences study design

All studies must disclose on these points even when the disclosure is negative.

Sample size	The number of biologically independent repeats is indicated in each legend or figure.
Data exclusions	n/a
Replication	For all statistical analysis data from at least two biological repeats performed on separate days was used. The exact number of replicates are presented in individual figure legends. Any differences in statistical significance are indicated.
Randomization	n/a
Blinding	n/a

Reporting for specific materials, systems and methods

We require information from authors about some types of materials, experimental systems and methods used in many studies. Here, indicate whether each material, system or method listed is relevant to your study. If you are not sure if a list item applies to your research, read the appropriate section before selecting a response.

Materials & experimental systems

n/a	Involved in the study	
☐	☒	Antibodies
☐	☒	Eukaryotic cell lines
☒	☐	Palaeontology and archaeology
☐	☒	Animals and other organisms
☒	☐	Human research participants
☒	☐	Clinical data
☒	☐	Dual use research of concern

Methods

n/a	Involved in the study	
☒	☐	ChIP-seq
☒	☐	Flow cytometry
☒	☐	MRI-based neuroimaging

Antibodies

Antibodies used	Polyclonal rabbit anti-HA antibody from A/Hong Kong/1073/99 was obtained from Sino Biological Inc., Beijing, China (A8592).<br>Goat anti-mouse IgG conjugated with horseradish peroxidase was obtained from Dingguo, Beijing, China (G8795).<br>Mouse monoclonal anti-GAPDH was obtained from Transgen (HC301).<br>Hybridoma cells secreting mouse monoclonal anti-influenza A virus NP (ATCC HB-65) was obtained from ATCC.
Validation	These antibodies are well established, commercially available, have been validated by the company and have been used by our group and many others. Polyclonal rabbit anti-HA antibody from A/Hong Kong/1073/99, Goat anti-mouse IgG conjugated with horseradish peroxidase, and mouse monoclonal anti-GAPDH antibodies were validated by the manufacturer by Western blot. monoclonal anti-influenza A virus NP was validated by the manufacturer by immunocytochemistry.

Eukaryotic cell lines

Policy information about cell lines

Cell line source(s)	HEK293T - human embryonic kidney<br>A549 - human lung epithelial<br>MDCK - Madin-Darby canine kidney<br>CEF - chicken embryo fibraoblasts<br>Vero - Vero cell<br>DF-1 - chicken fibroblast DF-1<br>All the cells were provided by the National Avian Influenza Para-Reference Laboratory (Guangzhou) at South China Agricultural University and have been used by our group and many others.
Authentication	RNA samples from each cells were deep sequenced by RNAseq and confirmed.
Mycoplasma contamination	All cell lines tested negative for mycoplasma contamination.
Commonly misidentified lines (See ICLAC register)	n/a

Animals and other organisms

Policy information about studies involving animals; ARRIVE guidelines recommended for reporting animal research

Laboratory animals	5-week-old SPF chickens were obtained from Guangdong Dahuanong Animal Health Products Co., Ltd., Guangdong Province, China; 4-week-old female BALB/c mice, obtained from the Vital River Company in Beijing, China.
Wild animals	n/a
Field-collected samples	n/a
Ethics oversight	All experiments were conducted in an animal biosafety level 3 laboratory and animal facilities at South China Agricultural University (SCAU) (CNAS BL0011) in accordance with protocols. All animals involved in the experiments were reviewed and approved by the Institution Animal Care and Use Committee at SCAU and treated in accordance with the guidelines (2017A002).

Note that full information on the approval of the study protocol must also be provided in the manuscript.
