# Supplementary material for: A risk marker of tribasic hemagglutinin cleavage site in influenza A (H9N2) virus
Source: Commun Biol. 2021 Jan 15;4:71. doi: 10.1038/s42003-020-01589-7 (PMC7811019; doi:10.1038/s42003-020-01589-7)
Supplement: Supplementary file 1 — Peer Review File [file 42003_2020_1589_MOESM1_ESM.pdf]

Reviewers' comments:

Reviewer #1 (Remarks to the Author):

H9N2 avian influenza viruses have spread worldwide in poultry, posing a threat to public health as well as the poultry industry. In this manuscript, amino acid sequences of the hemagglutinin (HA) cleavage site (HACS) of H9N2 influenza viruses sampled at different times and locations were investigated and 5 main cleavage site motifs were found, including PARSSR/G-, PSRSSR/G-, PAKSSR/G-, PAKSKR/G-, and PAASDR/G. The authors generated H9N2 viruses having HAs with these cleavage sites using a reverse genetics approach and compared biological properties of the viruses. They found that the tribasic H9N2 virus (i.e., a virus with the PAKSKR/G motif) had higher replication capacity in vitro and in vivo. Higher pH stability and thermostability were also observed in the tribasic H9N2 virus. In the tribasic H9N2 virus but not in the other viruses tested, the stem-loop structure of the RNA region that encodes the tribasic motif (PAKSKR), had an enlarged loop which has been shown to be important for nucleotide insertions at this region. This manuscript has interesting data and would provide important insights into the field. However, there are several weaknesses in its current state. Following are points that may improve the manuscript.

Major comments.

1. Figures 3a and 3b. The growth curve experiments were conducted in the presence of trypsin. It could be assumed that the addition of basic amino acid residues at HACS might

affect the cleavability of HA0, resulting in the increased viral titers. In other words, the difference in growth titers might be simply due to the difference in susceptibility of different HACS to host proteases. Thus, it is interesting to see the growth ability of the tribasic H9N2 virus in the absence of trypsin.

2. Figures 3d and 3e. The authors determined the effect of trypsin on the HA0 cleavage and concluded that there was no significant difference in the cleavability between rV08-PSRSSR and rV08-PAKSKR viruses. However, it should be important to see the cleavability at different concentrations of trypsin. Additional experiments are needed for this conclusion.

3. Figure 5. In this reporter assay, only luciferase activity was shown. It would also be important to show the sequence changes (i.e., actual nucleotide insertions) of the expressed RNAs in the cells or virions. Although the authors mention, "using Sanger sequencing, the single- and double-nucleotide insertions or double-nucleotide deletion occurred in the synthetize of mRNA or vRNA in the enlarged stem-loop structure of HACS (Fig. S5)", detailed methods of this sequencing analysis are missing. Explanation of Fig. S5 is also unclear. For example, what do the numbers in the parenthesis represent (e.g., 10/10)? How many percent of the viral RNAs contained the insertion(s)?

4. In Figure 5, the authors showed the stem-loop structure with 27nt sequences. However, it is not appropriate to generate secondary structures using just one type of software. The authors should use several different types of software to confirm the prediction of the RNA structures. In addition, the analyses using longer sequences (e.g., 40 and 50nt) should also be carried out.

Minor comments.

1. Some terms repeatedly used in the text, such as monobasic and tribasic, should be explained briefly.

2. Abstract. Rephrase "stem-loop RNA structures of HACS".

3. Line 83. The authors have referred to the P2, P4, and P5 positions (line 83). These positions could be shown in Figure 1 (box at the bottom) to make it easy for non-influenza researchers.

4. Figures 1b and 1c do not match the respective explanation in the text (page 6, lines 87-90). Similarly, Web logos (d and e) description does not match that of b and c (page 6, line 85). These should be carefully corrected in the text.

5. Figures 1b and 1c could be the same size.

6. Figure 1b-e. Additionally, the authors could insert titles such as "China", "All except China" so that the readers can read the figures easily.

7. Figure 2 legend symbol. These motif sequences should be consistent throughout the manuscript. For example, replace "ATSGR/GLF" with "PASTGR/G", etc., in Figure 2.

8. Figure 3c. The procedure for determining the thermal stability of the viruses is not clear. According to the figure legend, the viruses were inoculated for 1, 2, 3, 4, and 5 h at 50°C. Is this true? Should this be "incubated for 1, 2, 3, 4, and 5 h at 50°C"? If so, the x axis needs to be corrected.

9. "As expected, compared to the enlarged loop structure of 29-rV08-PAKSKR and 29-rV08-PAKKKR, the significantly lower luciferase expression was observed in the DF-1 cells when transfected with 29-rV08-PAKSKR-NL and 29-rV08-PAKKKR-NL plasmids" (Page 17, lines

269-271). This sentence should be modified since the difference between Link29-PAKSKR and Link29-PAKSKR-NL is not significant in Figure 5a.

10. Figure 6. Correct "RAKSKR" to "PAKSKR".

11. The title of Table S1. "Virus titers in chicken cloacal and oropharyngeal swabs" should be changed. Actually, this table does not show virus titers, it only shows infection rate, i.e., infected/total chickens.

12. The order of supplementary figures is strange in the text. Figure S1 is cited on line 312 after Figure S2-S6. It should be re-arranged.

13. Figures S2 and S3. How did the authors select these low pathogenic H5 and H7 strains? Are these limited to low pathogenic avian influenza viruses isolated from ducks or chickens?

14. Line 467. Provide a reference of "pHW2000"

15. Lines 560-562. Provide a reference of "ViennaRNA package".

16. There are many grammatical errors throughout the manuscript.

Reviewer #2 (Remarks to the Author):

Review report

A novel marker of triabasic hemagglutinin cleavage site in influenza A (H9N2) virus

Influenza A viruses (subtype H9N2) are prevalent in poultry in many countries and there are reports of occasional transmission to humans which is indicative of their pandemic potential. Cleavage of the surface glycoprotein hemagglutinin (HA) of H9N2 by host cell proteases at a distinct site is the central to H9N2 infections.

The authors analysed variations observed in the hemagglutinin cleavage site (HACS) of H9N2 using an array of computational & experimental approaches to evaluate replicate advantages, if any. The authors report that tribasic HACS in H9N2 are evolving to be hydrophilic and therefore provide distinct advantage to the virus for replication, stability, pathogenicity as well as transmission. The authors have made a case for how enlarged structure of RNA facilitate insertion without having any impact replication. It is definitely a well-structured study reported as a concise story.

I recommend that the authors come up with an info graphic summary this work to pictorially summarise various cleavage sites along side of assessment criteria (pH, temperature etc) summarising advantages/limitations. It would certainly add value to the manuscript.

Recommended edits:

1. The manuscript, however requires a revision to make it concise, especially in the results & methods sections. There are many mentions of the background knowledge & experimental details in the results section, which needs be avoided. For example, lines 140-142 on page 9. Please check all subsections in Results & revise suitably. Also every subsection begins

with a brief description the feature/parameter which is not necessary and is increasing the length of manuscript unnecessarily. For example, lines 138-140.

2. Page 5 lines 66-67: The statement, ‘Here we performed genetic evolution..’ Please redraft.

3. Page 9 lines 143-144: The statement: ‘Obviously increased infection’.. please replace obviously with ‘significantly’ and ‘obviously showed less …’ with ‘observed to be less’.

4. Page 20 line 309: The statement: ‘P6 and P1 positions were more stable than..’ please replace with ‘P6 and P1 positions were stable as compared to’.

5. Page 20 line 311: The statement: ‘However, there was a tendency to mutate to more hydrophobic residues in the P4 and P3’. Suggested revision: ‘Substitutions with hydrophobic residues were observed in the P4 and P3’.

6. Mention of SARS-CoV-2 harbour tribasic cleavage site is fine in the discussion section (page 22, lines 352-353 but needs to be excluded from the abstract.

Reviewers comments:

Reviewer #1 (Remarks to the Author):

H9N2 avian influenza viruses have spread worldwide in poultry, posing a threat to public health as well as the poultry industry. In this manuscript, amino acid sequences of the hemagglutinin (HA) cleavage site (HACS) of H9N2 influenza viruses sampled at different times and locations were investigated and 5 main cleavage site motifs were found, including PARSSR/G-, PSRSSR/G-, PAKSSR/G-, PAKSKR/G-, and PAASDR/G. The authors generated H9N2 viruses having HAs with these cleavage sites using a reverse genetics approach and compared biological properties of the viruses. They found that the tribasic H9N2 virus (i.e., a virus with the PAKSKR/G motif) had higher replication capacity in vitro and in vivo. Higher pH stability and thermostability were also observed in the tribasic H9N2 virus. In the tribasic H9N2 virus but not in the other viruses tested, the stem-loop structure of the RNA region that encodes the tribasic motif (PAKSKR), had an enlarged loop which has been shown to be important for nucleotide insertions at this region. This manuscript has interesting data and would provide important insights into the field. However, there are several weaknesses in its current state. Following are points that may improve the manuscript.

Major comments.

1. Figures 3a and 3b. The growth curve experiments were conducted in the presence of trypsin. It could be assumed that the addition of basic amino acid residues at HACS

might affect the cleavability of HA0, resulting in the increased viral titers. In other words, the difference in growth titers might be simply due to the difference in susceptibility of different HACS to host proteases. Thus, it is interesting to see the growth ability of the tribasic H9N2 virus in the absence of trypsin.

Response: Thank the reviewers for their constructive comments and valuable recommendations. We have assessed the growth ability of the dibasic H9N2 viruses (PARSSR/G-motif) and tribasic H9N2 viruses (PAKSKR/G-motif) in the absence of trypsin; however, we found that H9N2 viruses could not be efficiently cleaved and replicated. For low pathogenic H9N2 viruses, the HA0 precursor must be cleaved into the HA1 and HA2 subunits with the help of host protease, thereby revealing a fusion peptide for membrane fusion and replicate in the cells. In addition, many other studies proved that low pathogenic H9N2 viruses could not cleave and replicate in the avian and mammalian cells in the absence of the host protease.

2. Figures 3d and 3e. The authors determined the effect of trypsin on the HA0 cleavage and concluded that there was no significant difference in the cleavability between rV08-PSRSSR and rV08-PAKSKR viruses. However, it should be important to see the cleavability at different concentrations of trypsin. Additional experiments are needed for this conclusion.

Response: Authors agree with the reviewer. We have determined the cleavage efficiency of H9N2 viruses at different concentrations (0, 0.2, 0.4, 0.6, 0.8, and 1.0 μg/ml) of TPCK-treated trypsin in the CEF cells. However, we found that no significant

difference in the cleavability between rV08-PSRSSR and rV08-PAKSKR viruses in CEF cells in TPCK-treated trypsin at the concentrations of 0.4, 0.6, 0.8, and 1.0 μg/ml. We have added this results in the results and supplementary Figure section; please see ***page 9 lines 130-132 and Supplementary Figure 1***.

3. Figure 5. In this reporter assay, only luciferase activity was shown. It would also be important to show the sequence changes (i.e., actual nucleotide insertions) of the expressed RNAs in the cells or virions. Although the authors mention, "using Sanger sequencing, the single- and double-nucleotide insertions or double-nucleotide deletion occurred in the synthetize of mRNA or vRNA in the enlarged stem-loop structure of HACS (Fig. S5)", detailed methods of this sequencing analysis are missing. Explanation of Fig. S5 is also unclear. For example, what do the numbers in the parenthesis represent (e.g., 10/10)? How many percent of the viral RNAs contained the insertion(s)?

Response: Authors agree with the reviewer. We have added the detailed methods of this sequencing analysis and the sequence changes of expressed RNAs in the cells; please see ***pages 17-18 lines 269-276; page 36 lines 562-572***.

4. In Figure 5, the authors showed the stem-loop structure with 27nt sequences. However, it is not appropriate to generate secondary structures using just one type of software. The authors should use several different types of software to confirm the prediction of the RNA structures. In addition, the analyses using longer sequences (e.g.,

40 and 50nt) should also be carried out.

Response: Authors agree with the reviewer. We use several different types of software (RNAfold, mfold, and RNAstructure) and long RNA sequences (40 nt) to confirm the prediction of the RNA structures; please see ***supplementary Figures 5, 6, and 7***.

Minor comments.

1. Some terms repeatedly used in the text, such as monobasic and tribasic, should be explained briefly.

Response: Authors agree with the reviewer. We have added the description of the monobasic, dibasic, and tribasic in the ***introduction*** section; please see ***page 4 lines 50-52***.

2. Abstract. Rephrase "stem-loop RNA structures of HACS".

Response: Authors agree with the reviewer. We have rephrased the sentence. "the enlarged stem-loop structures of HACS in the RNA region were found in increasing tribasic H9N2 viruses"; please see ***page 1 lines 14-16***.

3. Line 83. The authors have referred to the P2, P4, and P5 positions (line 83). These positions could be shown in Figure 1 (box at the bottom) to make it easy for non-influenza researchers.

Response: Authors agree with the reviewer. We have added a mark at the bottom of ***Figure 1***; please see ***Figure 1***.

4. Figures 1b and 1c do not match the respective explanation in the text (page 6, lines 87-90). Similarly, Web logos (d and e) description does not match that of b and c (page 6, line 85). These should be carefully corrected in the text.

Response: Authors agree with the reviewer. We have checked it carefully and revised the sentences; please see ***page 6 lines 86, 88, and 89***.

5. Figures 1b and 1c could be the same size.

Response: Authors agree with the reviewer. We have revised the Figure 1b and 1c with the same size; please see ***Figure 1b and 1c***.

6. Figure 1b-e. Additionally, the authors could insert titles such as “China”, “All except China” so that the readers can read the figures easily.

Response: Authors agree with the reviewer. We have inserted the titles of “China” and “All except China” in the Figure 1b-e; please see ***Figure 1b-e***.

7. Figure 2 legend symbol. These motif sequences should be consistent throughout the manuscript. For example, replace “ATSGR/GLF” with “PASTGR/G”, etc., in Figure 2.

Response: Authors agree with the reviewer. We have replaced these motif sequences to make it consistent throughout the manuscript; please see ***Figure 2***.

8. Figure 3c. The procedure for determining the thermal stability of the viruses is not

clear. According to the figure legend, the viruses were inoculated for 1, 2, 3, 4, and 5 h at 50°C. Is this true? Should this be "incubated for 1, 2, 3, 4, and 5 h at 50°C"? If so, the x axis needs to be corrected.

Response: Authors agree with the reviewer. The word "inoculated" have been changed to "incubated"; please see ***page 48 line 755*** **and** ***Figure 3c***.

9. "As expected, compared to the enlarged loop structure of 29-rV08-PAKSKR and 29-rV08-PAKKKR, the significantly lower luciferase expression was observed in the DF-1 cells when transfected with 29-rV08-PAKSKR-NL and 29-rV08-PAKKKR-NL plasmids" (Page 17, lines 269-271). This sentence should be modified since the difference between Link29-PAKSKR and Link29-PAKSKR-NL is not significant in Figure 5a.

Response: Authors agree with the reviewer. We have revised the sentence; "the significantly lower luciferase expression was observed in the DF-1 cells when transfected with 29-rV08-PAKKKR-NL plasmids; however, the difference between 29-rV08-PAKSKR and 29-rV08-PAKSKR-NL is not significant (Fig. 5a)"; please see ***page 17 lines 265-267.***

10. Figure 6. Correct "RAKSKR" to "PAKSKR".

Response: Authors agree with the reviewer. We have corrected "RAKSKR" to "PAKSKR"; please see ***Figure 7***.

11. The title of Table S1. “Virus titers in chicken cloacal and oropharyngeal swabs” should be changed. Actually, this table does not show virus titers, it only shows infection rate, i.e., infected/total chickens.

Response: Authors agree with the reviewer. We have changed the titles to “The infection rate in chicken cloacal and oropharyngeal swabs”; please see ***Table S1***.

12. The order of supplementary figures is strange in the text. Figure S1 is cited on line 312 after Figure S2-S6. It should be re-arranged.

Response: Authors agree with the reviewer. We have re-arranged the orders of supplementary figures.

13. Figures S2 and S3. How did the authors select these low pathogenic H5 and H7 strains? Are these limited to low pathogenic avian influenza viruses isolated from ducks or chickens?

Response: Authors agree with the reviewer. The HACS sequences of low pathogenic H5, H7, and H9 precursors were all avian species (chickens, ducks, and wild birds) available in the GISAID database (http://www.gisaid.org/). Identical HACS sequences were removed. We have described more detail in the ***Methods section***; please see ***page 37 lines 578-580***.

14. Line 467. Provide a reference of “pHW2000”

Response: Authors agree with the reviewer. We have added a reference of “pHW2000”;

please see ***page 30 line 469***.

15. Lines 560-562. Provide a reference of “ViennaRNA package”.

Response: Authors agree with the reviewer. We have added a reference of “ViennaRNA package”; please see ***page 37 line 578***.

16. There are many grammatical errors throughout the manuscript.

Response: Authors agree with the reviewer. We have sought Dr. Matt T. from EditMyEnglish (http://www.editmyenglish.com) to proofread our entire manuscript. We have revised and corrected the errors carefully.

Reviewer #2 (Remarks to the Author):

Review report

A novel marker of triabasic hemagglutinin cleavage site in influenza A (H9N2) virus

Influenza A viruses (subtype H9N2) are prevalent in poultry in many countries and there are reports of occasional transmission to humans which is indicative of their pandemic potential. Cleavage of the surface glycoprotein hemagglutinin (HA) of H9N2 by host cell proteases at a distinct site is the central to H9N2 infections.

The authors analysed variations observed in the hemagglutinin cleavage site (HACS) of H9N2 using an array of computational & experimental approaches to evaluate

replicate advantages, if any. The authors report that tribasic HACS in H9N2 are evolving to be hydrophilic and therefore provide distinct advantage to the virus for replication, stability, pathogenicity as well as transmission. The authors have made a case for how enlarged structure of RNA facilitate insertion without having any impact replication. It is definitely a well-structured study reported as a concise story.

I recommend that the authors come up with an info graphic summary this work to pictorially summarise various cleavage sites along side of assessment criteria (pH, temperature etc) summarising advantages/limitations. It would certainly add value to the manuscript.

Response: Authors agree with the reviewer. We have added a graphic summary to summarise various cleavage sites along side of assessment criteria summarising advantages/limitations. please see ***Figure 9***.

Recommended edits:

1. The manuscript, however requires a revision to make it concise, especially in the results & methods sections. There are many mentions of the background knowledge & experimental details in the results section, which needs be avoided. For example, lines 140-142 on page 9. Please check all subsections in Results & revise suitably. Also every subsection begins with a brief description the feature/parameter which is not necessary and is increasing the length of manuscript unnecessarily. For example, lines 138-140.

Response: Authors agree with the reviewer. We have revised the background knowledge & experimental details in the ***result section***. In addition, we have deleted the brief description the feature/parameter in the ***result section***.

2. Page 5 lines 66-67: The statement, ‘Here we performed genetic evolution..’ Please redraft.

Response: Authors agree with the reviewer. We have redrafted the statement, “Here we performed genetic evolution” to “In this study, we analyze the genetic evolution…”; please see ***page 5 line 69***.

3. Page 9 lines 143-144: The statement: ‘Obviously increased infection’.. please replace obviously with ‘significantly’ and ‘obviously showed less …’ with ‘observed to be less’.

Response: Authors agree with the reviewer. We have revised these sentence; please see ***page 9 lines 139-141***. “In contrast to the other viruses, the rV08-PAKSKR virus displayed a significantly increased infectious titer after incubation at each time point compared with the other H9N2 mutants. In contrast, the rV08-PAASDR virus were observed to be less stable than the parental virus at each time point.”

4. Page 20 line 309: The statement: ‘P6 and P1 positions were more stable than..’ please replace with ‘P6 and P1 positions were stable as compared to’.

Response: Authors agree with the reviewer. We have revised the sentence; please see ***page 20 line 309.*** “we found that the P6 and P1 positions were stable as compared to

other residues during the viral evolution".

5. Page 20 line 311: The statement: 'However, there was a tendency to mutate to more hydrophobic residues in the P4 and P3'. Suggested revision: 'Substitutions with hydrophobic residues were observed in the P4 and P3'.

Response: Authors agree with the reviewer. We have revised the sentence; please see ***page 20 line 310***. "substitutions with hydrophobic residues were observed in the P4 and P3".

6. Mention of SARS-CoV-2 harbour tribasic cleavage site is fine in the discussion section (page 22, lines 352-353 but needs to be excluded from the abstract.

Response: Authors agree with the reviewer. We have deleted the mention of SARS-CoV-2 in the abstract section.

We hope that our revised version will be satisfactory for you and the reviewers. Great thanks to you and the referee for the time and efforts you expend on our manuscript.

Yours Sincerely,

Dr. Wenbao Qi,

South China Agricultural University, Guangzhou, P.R. China.

REVIEWERS' COMMENTS:

Reviewer #2 (Remarks to the Author):

The authors have satisfyingly addressed all the previous comments raised by this reviewer. Concerns from the other reviewer's are also well answered.

Reviewer #3 (Remarks to the Author):

Review of “Circulating miR-424 loaded extracellular vesicles are oncogenically active in experimental models and prostate cancer patients”

Following my previous comments, the revised manuscript is well written and much easier to follow now.

I have two recommendations

Line 141-142

Specifically here, the functionality of miR-424 within EVs was not tested to claim that miR-424 was 'fully functional'. Could the authors reword the sentence to something along the lines of:

"Importantly, miR-424 was internalised within EVs, protected by the EV phospholipid membrane from degradation, as shown by its resistance to RNase treatment".

Previous comment: EV characterisation - In section 4 of the MISEV2018 checklist, it is recommended that two EV quantitation methods are used (eg. Particle and protein) and presented as a ratio of the two quantitation figures.

If data is available for the EV protein concentrations and it is possible, add the protein concentrations to Figure 1, with a figure showing the ratio of the NTA count vs protein concentration
